# Preclinical characterization of an active immunotherapy targeting calcitonin gene-related peptide

**DOI:** 10.1038/s43856-025-00870-2

**Published:** 2025-04-29

**Authors:** Justin D. Boyd, Shixia Wang, Hsiao-Wen Lin, Yueh-Ting Hsieh, Yu Shuang Sun, Brett A. Thibodeaux, Hanxin Lu, Jaya Sahni, Jonathan Wiggins, Matthew S. Longo, Jeanne K. Brooks, Madeline M. Vroom, Yi-Pin Chang, Zhi Liu, Shuang Ding, Jean-Cosme Dodart

**Affiliations:** Vaxxinity Inc., Merritt Island, FL USA

**Keywords:** Neurology, Drug discovery

## Abstract

**Background:**

The success of passive immunotherapies targeting Calcitonin gene-related peptide (CGRP) for managing migraine has prompted our efforts towards developing an active immunotherapy that induces the production of endogenous antibodies against CGRP. Achieving efficacious antibody titers via immunization could provide a more convenient and cost-effective treatment alternative to anti-CGRP monoclonal antibody (mAb) therapies. However, immunization against endogenous CGRP faces multiple challenges such as breaking immune tolerance, inducing sufficient antibody titers, and avoiding immune response-associated toxicity.

**Methods:**

Synthetic peptide immunogens formulated in adjuvants were delivered intramuscularly. Serum samples were collected post immunization and used to measure antibody titers as well as for the isolation of antibodies specific to CGRP. Antibodies were characterized for their binding affinities and specificities. The capsaicin-induced increase in dermal blood flow model was used in rats for the assessment of the pharmacodynamic effect of immunization.

**Results:**

Here we demonstrate that a peptide-based active immunotherapy designed to induce antibodies against CGRP promotes robust antibody titers across preclinical species. Characterization of the immune response strongly suggests that this peptide immunogen primarily stimulates a humoral response and only induced CGRP-specific antibodies. Antibodies produced by immunization are primarily IgG1 and demonstrate binding and activity potencies similar to marketed monoclonal antibodies against CGRP. Finally, immunization demonstrates in vivo efficacy in a rat pharmacodynamic model.

**Conclusion:**

Our results strongly suggest that a peptide-based active immunotherapy against CGRP could provide an affordable and convenient therapeutic for the prevention of migraine.

## Plain language summary

People who have migraines experience recurrent headaches that are often also accompanied by nausea, vomiting, and sensitivity to sound and light. We developed a possible treatment for migraine that targets a peptide known to contribute to migraine episodes, called human calcitonin gene related peptide (CGRP). Our treatment consistently induced the production of antibodies able to identify and neutralize CGRP in rats and non-human primates. These results were similar to those seen with other treatments that target CGRP, suggesting our treatment might have beneficial effects in people with migraines. Our treatment might be a potential alternative to current migraine treatments.

## Abstract (web summary)

Boyd, Wang et al. evaluate a peptide-based active immunotherapy against CGRP in rat and non-human primate models. The immunotherapy consistently induces robust IgG1 antibodies that exhibit potent in vitro binding and blocking activity against CGRP and in vivo efficacy in a rat pharmacodynamic model.

## Introduction

Migraine is a disabiliting disorder, which consists of a unilateral headache often accompanied by nausea, vomiting, and sensitivity to sound and light. It mostly occurs in young adults and middle-aged women, and it has been identified by the global burden of disease^1,2^ as the sixth most prevalent disorder. The pathophysiology of migraine involves changes in the vasculature, central and peripheral pain processing, and inflammation^3–5^. Calcitonin gene-related peptide (CGRP) plays an important role in the pathophysiology of migraine. An increase in CGRP is observed during acute migraine attacks, and efficacious sumatriptan treatment normalizes CGRP levels. Moreover, an intravenous injection of CGRP induces migraine-like attacks in migraine patients. These observations suggested that targeting the peptide or its receptor could have potential as migraine therapies^6^.

CGRP and its receptor are expressed in both peripheral nervous system (PNS) and central nervous system (CNS). CGRP is released from trigeminal afferent nerve fibers during a migraine and causes vasodilatation and neurogenic inflammation^7,8^. Both small molecule and biologics have been successfully developed for the prevention of migraine episodes. Small molecules known as gepants block the CGRP receptor. Monoclonal antibodies (mAbs) targeting either CGRP (Fremanezumab, Eptinezumab, and Galcanezumab) or its receptor (Erenumab) have been developed and approved by the United States Food and Drug Administration (USFDA) and the European Medicines Agency (EMA) for the prevention of episodic and chronic migraine^9^. These mAbs have proven effective in migraine subjects that previously failed other preventive therapies^10^. Importantly, functional blockade of CGRP with mAbs has also demonstrated good safety profiles due to their high target specificity.

The effectiveness and safety profile of mAbs targeting CGRP prompted us to develop an active immunotherapy against CGRP, which could provide a more accessible and convenient alternative to mAbs for migraineurs. Ideally, an active immunotherapy targeting CGRP would stimulate a safe humoral response and show similar efficacy as mAbs. Here, we describe a peptide immunogen comprising a CGRP-based B cell epitope linked to a T Helper (T_H_) peptide that induced robust antibodies against endogenous CGRP in preclinical species (rats, guinea pigs, and macaques). Importantly, we observe no evidence of overt inflammation, nor do we observe stimulation of immune response by endogenous CGRP following immunization. This peptide immunogen induced anti-CGRP antibodies, enriched for IgG1, that demonstrated binding and functional properties comparable to mAbs. Moreover, an anti-CGRP peptide immunogen targeting rat CGRP demonstrated effects comparable to mAbs in a pharmacodynamic rat model. These findings provide the basis for developing an active immunotherapy targeting CGRP in people living with migraine.

## Methods

### CGRP peptide sequence analysis

Sequences for CGRP peptide were sourced from the National Center for Biotechnology Information (NCBI) for a comparative analysis between humans, non-human primates (Cynomolgous monkeys), rats, and guinea pigs. Several sequences were included for each species from which the 37-residue-long region of interest was manually selected. A MUSCLE multiple sequence alignment was run via EMBL-EBI to identify species-specific variations. A representative alignment was subsequently manually constructed to highlight sequence similarity relative to human αCGRP. Moreover, the Immune Epitope Database (IEDB) confirmed the absence of T cell epitopes in our selected sequences.

### Peptide immunogens and antigens

The C-terminus epitope (aa28-37) of human α-CGRP (hCGRP_28-37_, p4796a) and rat α-CGRP (rCGRP_28-37_, p5830a) were designed as B cell epitopes to induce specific antibody responses. The individual B cell epitope peptide without C-terminal amide was synthetically linked to the C-terminus of a proprietary T helper (T_H_) peptide MvF5-Th (US patent no. 9,102,752) to generate the peptide immunogens T_H_-hCGRP_28-37_ (p4796kb) and T_H_-rCGRP_28-37_ (p5830kb) for immunogenicity studies in animals. The full-length human α-CGRP_1-37_ (hCGRP_1-37_, ACDTATCVTHRLAGLLSRSGGVVKNNFVPTNVGSKAF) and full-length rat α-CGRP_1-37_ (rCGRP_1-37_, SCDTATCVTHRLAGLLSRSGGVVKDNFVPTNVGSEAF), which contained the C-terminal amide, were used as antigen to detect the CGRP-specific antibody responses by ELISA (detailed methods described below). The peptides were either synthesized in house using automated solid-phase synthesis and were purified by preparative HPLC or commercially sourced from Bachem (CA, product number 4013281).

### Peptide immunogen formulations

A proprietary polyanionic CpG oligonucleotide (ODN, CpG1) was formulated with p4796kb or p5380kb peptides, at a 1.8:1 (peptide:CpG1) molar charge ratio. The peptide/CpG1 complexes were further formulated with either alum adjuvant (Adju-Phos®) or oil-in-water emission adjuvant (Montanide^TM^ ISA 51VG). Adju-Phos®-based formulations contained 1500 µg/ml of peptide(s), 445 µg/ml of CpG1 (peptide:CpG1 molar charge ratio as 1.8:1), and 1.6 mg/ml of Aluminum. The ISA 51VG-based formulations contained 1500 µg/ml of peptide(s), 445 µg/ml of CpG1 (peptide:CpG1 molar charge ratio as 1.8:1).

### In vivo immunogenicity studies

All animal studies were conducted by contract research organizations (LabCorp or Charles River Laboratories). Animals were bred and used in accordance with protocols approved by their internal Institutional Animal Care and Use Committee (IACUC). All attempts to minimize animal numbers were taken and reflected in species used for each study. Additionally, repeated in-life blood collection frequency and volumes comply with the NIH guidelines for survival blood collection. Humane endpoints were considered in all studies if an animal was found displaying labored breathing, moribund, had ataxia that prohibits access to food and water, paralysis, had lost 20% or more of its body weight, or other (at the discretion of the veterinarian). However, humane endpoints were never reached in any of the studies presented here. Guinea pigs and rats were used in immunogenicity studies to allow sufficient blood collection volumes for antibody purification and characterization. Monkeys were used to confirm immunogenicity of the peptide immunogens in a larger species with close homology to humans. Most studies included both males and females in an equal ratio; the exception to this rule occurred when animal availability from the supplier was limited or for the pharmacodynamic rat model where only males were used (see below). For in vivo studies involving multiple treatment groups, animals were randomly assigned to the study using a computerized procedure designed to achieve body weight balance across groups. Experimenters blind to the treatment status of the animals performed the injections and conducted the in vivo experiments.

Duncan-Hartley Guinea pigs were used for immunogenicity studies and to characterize immunization-derived antibodies. Guinea pigs (*n* = 3 males; 300–350 g; 6–8 week-old) were immunized with adjuvanted p4796kb peptide by intramuscular (IM) injections administered at Weeks 0, 3, and 6 (40 μg peptide/dose). Blood samples were collected by orbital vein puncture prior to each dose (only once every 3 weeks and alternating eyes between bleeds) and 3 weeks after the last dose. For survival blood collection, Guinea pigs were sedated with Ketamine (1–8 mg/kg, intramuscular injection), a micro-capillary tube (non-heparinized) was inserted on an angle into the space between the globe and lower eyelid at the corner of the eye, and a maximum volume of 1–2 mL of whole blood was collected. At completion of the study, following a lethal dose of Ketamine, a maximum volume of blood was collected by cardiac puncture. All blood samples were processed into serum and kept at −80 °C until antibody titer analyses.

Four rat immunization studies were conducted to evaluate the immunogenicity, antibody properties, and effects of various CGRP peptide immunogen regimens. For all studies, survival blood collection volume was 1–2 mL of whole blood collected by orbital vein puncture as described above. At completion of each study, following a lethal dose of Ketamine, a maximum volume of blood was collected via cardiac puncture. Blood samples were processed into serum and kept at -80 °C until antibody titer analyses. In the first study, Wistar rats (*n* = 3 males and 3 females per group, 2–3 month-old, 250–300 g) were immunized with 30 μg/dose p4796kb in Adju-Phos® and CpG1 (IM) at days 1, 22, 43, and 64. Serum samples were collected every three weeks, and serum titers analyses were conducted as indicated below. The second study was conducted in Sprague–Dawley rats. Male rats (*n* = 5 per group, 2–3 month-old, 150–250 g) were immunized with 300 µg of peptide/dose with p4796kb formulated in Adju-Phos® or ISA 51VG. Additionally, there were two adjuvant control groups that received CpG1+Adju-Phos® or CpG1+ISA 51VG. The immunizations were performed on Days 0, 21, 42, 56, and 84. The sera were collected on Day -2, on Days 0, 21, 42, 56, and 84 at 6 h after dosing to evaluate the cytokines response, and on Days 14, 35, 58, 79, and 105 to evaluate the antibody responses. The third study was conducted in Sprague Dawley rats (*n* = 5 males and *n* = 5 females per group, 11-12 week-old, 250–450 g), animals were injected with saline, Adju-Phos®, p4796kb at 300 μg, or p5830kb at 300 μg on Days 2, 16, 30, 44, 58, and 72. The sera were collected on Days 2, 16, 30, 44, 58, 72, and 184. For the fourth rat study, Sprague Dawley rats (*n* = 6 in the p5830kb group and *n* = 8 per group in the other groups; 8 week-old; 200–300 g) received repeat IM injections of saline, Adju-Phos®, p4796kb at 300 μg, p5830kb at 300 μg on Days 1, 22, 43, 64, and 85. Only males were used in this study to avoid potential variable responses to capsaicin between genders. All peptides were formulated in Adju-Phos® and CpG in each dose group. An additional group of rats received a single intravenous infusion of Galcanezumab (5 mg/kg) on Day 85 as a positive control. Two weeks after the last injection (Day 99), rats were submitted to a capsaicin challenge (see methods below).

A Cynomolgus macaque study was conducted to further evaluate the immunogenicity of p4796kb in an Adju-Phos®/CpG1 formulation in non-human primates. Certified Primate Diet (PMI Nutrition International Certified LabDiet®) was provided once or twice daily. Greenfield city water was provided ad libitum. Water samples were routinely analyzed for specified microorganisms and environmental contaminants. Environmental controls for the animal room were set to maintain a temperature range of 20–26 °C, a relative humidity of 30–70%, a minimum of eight air changes/hour, and a 12 h light/12 h dark cycle. Animals were given various cage-enrichment devices and were acclimated to the environment for at least three weeks prior to the start of the experiment. Two groups of macaques (25–50 months, 2.5 to 5.5 kg, males, *n* = 6 per group) were immunized with p4796kb (300 µg peptide/dose, IM, 0.5 mL) or Vehicle control at weeks 0, 3, 6, 9, and 12. Blood samples (~1 mL) were collected from a femoral vein prior to dosing. Samples were processed into serum and kept at −80 °C until antibody titer analyses. All animals were placed in the stock colony at the conclusion of the study.

### Antibody titer assays

ELISA-based titer assays were conducted to evaluate the antibody response against CGRP in guinea pig, rat, and macaque serum samples. Microtiter 96-well plates were 1st coated with 100 μL of 2 μg/mL of the full-length synthetic human or rat αCGRP_1-37_ (Bachem, CA, product number 4013281) peptide at room temperature (RT) for 1 h and 4 °C overnight. Plates were washed 4 times with PBS-T to remove the coating solution and blocked with 10% bovine serum albumin in PBS. After blocking for 2 h, guinea pig, rat, or macaque serum samples were incubated in a 12-point half Log serial dilution for 1 h at RT. Plates were rinsed 4 times in 1x PBS-T. Horseradish Peroxidase (HRP)-conjugated Protein A/G 1:10,000 (ThermoFisher) was used to detect antibodies against CGRP in rat, guinea pig, and macaque sera. Species specific HRP-conjugated secondary antibodies were used for antibody isotyping at 1:5000 dilution: anti-rat IgG1-HRP (Southern Biotech 3060-05), anti-rat IgG2a-HRP (Southern Biotech 3060-05), anti-rat IgG2b-HRP (Southern Biotech 3070-05), anti-rat IgG2c-HRP (Southern Biotech 3075-05), anti-baboon IgG1 (Absolute Antibodies ab01619-23.0-1X200UG), anti-baboon IgG2 (Absolute Antibodies ab01620-23.0-1X200UG), anti-baboon IgG3 (Absolute Antibodies ab01621-23.0-1X200UG), anti-baboon IgG4 (Absolute Antibody ab01624-23.0-1X200UG). After incubation with HRP-conjugated Protein A/G or secondary antibody, 3,3’,5,5’-Tetramethylbenzidine (TMB) substrate was applied to each well for 15 min, and the reaction was stopped with 2 N sulfuric acid. Absorbance was measured at 450 nm on an iD5 plate reader (Molecular Devices, PA). A non-linear four-parameter curve fit was applied to each dilution curve to calculate the EC_50_ (SoftMax Pro, Molecular Devices, PA). To measure anti-CGRP antibody concentrations in rat sera, the same ELISA protocol as described above was used but an 8-point calibration curve (1.7-fold serial dilution from 100 ng/mL) was generated using a mouse monoclonal antibody against CGRP (Sigma Aldrich C7113). Here, microtiter 96-well plates were coated with 100 μL of 2 μg/mL of the full-length synthetic human αCGRP_1-37_. Anti-CGRP antibody concentrations were determined by interpolation from the calibration curve using a 4-PL nonlinear regression model.

### Dot blot assay

PVDF membranes were loaded onto dot blot cassettes (Bio-Rad, CA) and were activated using methanol at 100 μL/well. Methanol was then removed by suction. A CGRP coating solution was prepared by diluting synthetic human αCGRP stock in ddH2O to 2 μg/ml. 100 μL/well of CGRP was transferred to coat the membranes and removed by suction. Membranes were washed with 100 μL/well of ddH2O followed by suction. Membranes were then blocked with 100 μL/well of 5% BSA in PBST and kept on a rotating plate at room temperature (RT) for 1 h. Membranes were washed three times with 300 μL/well of PBST for a few seconds. Sample sera were diluted in 3% BSA in PBST by 100, 1000, and 10,000 fold. Next, 100 μL of diluted serum was added to each well and rotated for 2 h at RT. PVDF membranes were taken out of the dot blot cassette, put into plastic containers, and washed three times with PBST for 5 min. HRP-conjugated anti-guinea pig antibody (Jackson ImmunoResearch 706-035-148) was diluted 1:3000 in 3% BSA/PBST, and 5 mL was applied to the membranes. Membranes were incubated for 1 hour at RT on a rotating plate. Membranes were washed three times with PBST for 5 min. ECL substrate (Bio-Rad 170-5061) was then applied to the membranes. Chemiluminescent signals were captured using UVP image system (ThermoFisher). Images were analyzed using VisionWorks LS 8.2.

### Rat splenocytes IFNγ ELISpot

Splenocytes were collected from control rats and from immunized rats 3 weeks after the last injection and stored at −80 °C. Cell thawing reagents (FBS, heat-inactivated) were equilibrated with the complete cell culture medium (RPMI with glutamax +20% FBS + HEPES + β-mercaptoethanol; all GIBCO) at room temperature. Splenocytes were retrieved from -80 °C and thawed in a 37 °C water bath until a small piece of ice was left. Contents of the cryovial were transferred to 5 mL of HI-FBS (in a 50 mL tube) dropwise, while swirling. The volume in the tube containing HI-FBS and thawed contents was brought up to 40 mL with room temperature PBS and centrifuged at 300 x *g*, at RT for 5 min. Supernatant was removed by decanting and resuspended in 4–5 mL of complete media. Cells were counted and diluted to 1–2 million per well in a T-25 flask for a 4 h recovery in a cell culture incubator (37 °C, 5% CO2). Cells were then removed from the cell culture incubator and plated at 1 × 10^5^ cells/well in a pre-blocked IFNγ ELISpot^PLUS^ plate (MABTECH, Nacka Strand, Sweden). Splenocytes were stimulated in vitro with each individual peptide component of the peptide immunogen: T_H_ peptide, CGRP_28-37_ or full peptide immunogen at 5 µg/mL. Control splenocytes were stimulated either with medium alone (negative control) or 1 μg/mL ConA (positive control). For IFNγ detection, rat splenocytes were stimulated with individual peptides for 48 hours at 37 °C. Cells were then removed by emptying the plate and washed 5 times with 200 μL PBS. The detection anti-IFNγ mAb (MTAM8-biotin) was diluted to 1 μg/mL in PBS containing 0.5% fetal calf serum. 100 μL of mAb was added to each well and incubated for 2 h at room temperature. Wells were washed 5 times with 200 μL PBS. Streptavidin-HRP was diluted 1:1000 in PBS-0.5% FCS, 100 μL added to wells, and incubated 1.5 h at room temperature. Wells were rinsed 5 times with 200 μL PBS followed by the addition of the ready-to-use TMB substrate for resolving the spots. Upon the emergence of spots, the reaction was stopped by washing in deionized water. Plates were dried then imaged. Spots were quantified using an AID iSpot reader (Advanced Imaging Devices GmbH, Germany). Spot-forming units (SFU) per million cells were calculated by subtracting the negative control wells.

### Affinity purification of CGRP-specific IgG from immune sera

For antibody characterization, hyperimmune rat sera were collected post-immunization, pooled by group, and passed through a 0.2 μm filter. To isolate total IgG fractions, NAb A/G protein columns (Thermo Fisher Scientific) were washed with two resin volumes of Pierce Protein A/G Binding Buffer (Thermo Fisher Scientific). After filtration, sera were diluted two-fold in binding buffer and incubated with end-over-end mixing for one hour. The column was subsequently washed with two volumes of binding buffer, and bound antibodies were eluted four times in 5 mL of IgG Elution Buffer (GBiosciences) neutralized with 250 μL of Tris-HCl (pH 8.0, Anatrace). Triplicate IgG isolations were performed for each group. Total IgG fraction was concentrated to ≤ 2% of its original volume in a 100 kDa Amicon filter unit (MilliPoreSigma) with centrifugation at 6 °C and 4000 × *g*, then buffer exchanged into ultrapure DNase/RNase free distilled water (Invitrogen). The protein content of the total IgG fraction concentrate was determined using a QuBit Fluorometer 4.0 (Invitrogen) protein assay, per the manufacturer’s instructions.

For affinity purification of CGRP-specific antibodies, disposable columns were packed with 2 mL of streptavidin agarose slurry (Thermo Fisher Scientific) and stored in ultrapure DI supplemented with sodium azide to a final concentration of 0.02% at 2-8 °C. All materials and reagents were adjusted to room temperature prior to use, and the washes and elution were accomplished via gravity flow. To link the biotinylated peptides, the streptavidin columns were washed with five resin bed volumes of 1X PBS (R&D). Rat or human CGRP peptides were reconstituted in ultrapure DI, and up to 3 mg of total protein was applied to each column, respectively. The streptavidin slurry and biotinylated peptides were incubated overnight at 2–8 °C. The next day, each column was washed with five volumes of binding buffer. Total IgG fraction concentrate was diluted in binding buffer, applied to the column, and incubated in the resin bed overnight at 2–8 °C. The next day, each column was washed with five volumes of binding buffer, and bound anti-CGRP antibody was eluted as previously described. Anti-CGRP affinity purifications were performed in triplicates for each group. Fractions from each group were pooled and subsequently concentrated to ≤ 1% of its original volume in a 100 kDa Amicon filter unit (MilliPoreSigma) with centrifugation at 6 °C and 4000 × *g*, then buffer exchanged into ultrapure DNase/RNase free distilled water (Invitrogen). The protein content of the affinity purified antibody was determined using a QuBit Fluorometer 4.0 (Invitrogen) protein assay per the manufacturer’s instructions.

### Binding affinities of CGRP-specific antibodies

Monoclonal antibodies (Fremanezumab and Galcanezumab) and p4796kb antibodies were immobilized at 25 °C on CM5 chips with an amine coupling kit using standard amine coupling procedure: sensor chip was initially activated by 0.4 M of 1-ethyl-3-(3-dimethylaminopropyl)-carbodimide (EDC) mixed with 50% 0.1 M N-hydroxysuccinimide (NHS) at a flow rate of 10 μL/min for 7 min. Antibodies were diluted in immobilization buffers: Fremanezumab and p4796kb antibodies at 10 μg/mL in 10 mM Na Acetate at pH 4.0; Galcanezumab at 8 μg/mL in 10 mM Na Acetate at pH 4.5. Diluted antibodies were injected to flow cell 2 at a flow rate of 10 μL/min for 360 seconds in channels 4, 5, 6, 7, and 3, respectively. The remaining active sites on the sensor chips were blocked with 1 M ethanolamine-HCl solution, pH 8.5 at a flow rate of 10 μL/min for 7 min. The flow cells were washed with an injection of running buffer at 30 μL/min for 600 s before use. Antibodies were immobilized on a CM5 chip at 25 °C. Two-fold serial dilution of CGRP analyte (5 nM, 2.5 nM, 1.25 nM, 0.625 nM, and 0.3125 nM) was sequentially injected at 25 °C to channel 4 at a flow rate of 30 µl/min for 120 s followed by dissociation for 600 s. The curves were fitted with Biacore®8 K analysis software (BIAevaluation) using a 1:1 Langmuir binding model.

### Analyses of off-target binding of anti-αCGRP antibodies

Guinea pig sera collected at week 15 post p4796kb immunization were assessed for their potential off target binding using HuProt™ Human Proteome Microarrays v4.0 (Cambridge Protein Arrays Ltd., Cambridge, UK) with over 20,000 individual proteins, representing more than 16,000 human genes, including the peptides of the calcitonin/CGRP peptide family. Proteins were expressed in yeast (S. cerevisiae) as GST fusions, purified, and spotted in duplicate. The microarray further contained several additional control proteins including rhodamine+IgG 647 (rhodamine + Alexa Fluor 647 labeled IgG) as landmarks for fluorescence detection at wavelengths of 532 nm/635 nm. Incubation of a HuProt™ Microarray v4.0 with the p4796kb-immunized serum at a dilution of 1:1000 was followed by staining with the secondary antibody as well as read-out at a scanning intensity of 7 (red). Hits were further evaluated by epitope mapping microarray. The original antigen αCGRP (CALCA gene) and the top protein hits NDRG4, SRXN1, DCST1, SHCBP, SNX1, OGA, DHR13, ERIC2, BCAR1, TESK2, I2BP2, ES8L2, UBTD2, S23IP, HXK4, HS90B, VINC, 5NTC, RHG01, PACN2, MYL4, AK1BA, DP13B, NUD11, and PTPRD were linked and elongated with neutral GSGSGSG linkers at the C- and N-termini to avoid truncated peptides. The linked and elongated sequences were translated into 15-amino-acid peptides with peptide-peptide overlaps of 14 aa for αCGRP and 12 aa for all other proteins. The resulting peptide microarrays contained 4,919 different peptides printed in duplicate (9836 peptide spots) and were framed by additional HA control peptides (YPYDVPDYAG, 218 spots). Both pre-immune guinea pig serum (pooled from 3 GPs) and p4796kb-immunized GP serum (pooled from 2 GPs at 15 weeks post-immunization) were profiled in this microarray. Binding of p4796kb-derived sera to 3 hits from the HuProt™ microarray screen (HSP90 beta protein, StressMarq Bioscience, SPR-102B; AKR1B10 protein, MyBioSource, MBS203315; PTPRD protein, Acro, PTD-H52H9) and to other propeptides that belong to the calcitonin/CGRP peptide family, including recombinant adrenomedullin (ADM, MyBioSource, MBS2012013), recombinant adrenomedullin 2 (ADM2, MyBioSource, MBS2123890), synthetic amylin (Abcam, ab142398), and recombinant calcitonin (Abcam, ab153793), were further evaluated. For each peptide or protein, binding was assessed by using the same ELISA-based binding assays as described above. Microtiter 96-well plates were initially coated with 100 μL of 2 μg/mL of peptide or protein, and the plates were further processed as described above.

### CGRP-induced cAMP cell-based assay

CGRP-induced cAMP was assessed in SK-N-MC cells using the Bridge-It® Cyclic AMP assay according to manufacturer’s instructions (Mediomics, St. Louis, MO). Briefly, cells were passaged and rinsed in PBS before resuspending cells at 10,000 cell/μL in KRB-IBMX buffer. Serially diluted anti-CGRP antibodies were added to the samples and incubated for 20 min at room temperature. Synthetic CGRP (Bachem, Catalog No.4013281.1000) was then added to the samples at a final concentration of 100 nM and incubated for 60 min at room temperature on a shaker. All-in-one Assay Solution was added to samples to lyse cells and incubated for 1.5 h at room temperature. 18 μL from each sample was transferred to wells of a 384 well plate and read on a plate reader at 485 nm (excitation) and 540 nm (emission). Percent Activity was calculated using the formula: Percent Activity = [(Fx − Fneg)/(Fpos – Fneg)] x 100, where Fx = Fluorescence intensity of the sample, Fneg = Fluorescence intensity of the negative control (sample with CGRP), and Fpos = Fluorescence intensity of the positive control (sample without CGRP).

### Capsaicin-induced increase in dermal blood flow in rats

Laser Doppler imaging (LDI) was performed as described by Benschop et al^11^. Briefly, Sprague Dawley rats (*n* = 6 in the p5830kb group and *n* = 8 per group in all other groups) received repeat intramuscular injections of saline, Adju-Phos, p4796kb or p5380kb at 300 μg on Days 1, 22, 43, 64, and 85. This priming regimen was selected to maximize the chances of observing a pharmacodynamic effect and is based on previous experience indicating that maximal potency of antibodies is achieved within 9 to 15 weeks of immunization, despite that maximal antibody titers can be achieved within 3–6 weeks. An additional group of rats received a single intravenous infusion of Galcanezumab (5 mg/kg) on Day 85 as a positive control. On the day of the capsaicin challenge, rats were first anesthetized under 5% isoflurane in an induction chamber, then placed on a heating pad under the LDI instrument and stabilized under 1-2% isoflurane for 20 min prior to imaging. During this period, the abdomens of the rats were shaved to expose the skin. Two or three O-rings were placed on the shaved abdominal skin and approximately 1 cm apart from each other. O-rings were placed on areas without major vessels, as confirmed by scanning at a speed of 4 ms/pixel followed by 10 ms/pixel. Serial scanning with the LDI2-IR Laser Doppler Imager (Moor Instruments, Wilmington, DE) began with two baseline scans in a speed of 10 ms/pixel, followed by applying 8 μL of capsaicin solution (2 mg) for each o-ring. Scanning continued for an additional 25 min, and images were taken every 2.5 min. Blood flow images were analyzed using moorLDI Review V6.0 software (Wilmington, DE) in regions of interest, either areas inside the o-rings or the whole images.

### Statistical analyses

All statistical analyses were performed using Prism 9.0 v. 9.2.0 (Graphpad, San Diego, CA). Samples were assayed in replicates of two or three unless otherwise noted. For serum titer analyses, replicates were collected within the same experiment and across experiments (at least one replicate experiment on a different day). In the case of isotyping and ELISpot experiments, two experiments were conducted by different individuals to confirm reproducibility. For in vitro and in vivo assays, “biological” replicates are presented. For guinea pig (*n* = 3 per group) and NHP (*n* = 6 per group) studies, cohort sizes were chosen to minimize the number of animals while providing statistical robustness. For the rat in vivo studies, 6–8 animals per cohort were selected to minimize the number of animals used while providing sufficient statistical power, as well as sufficient serum volume for antibody and cytokine characterization and splenocytes for ELISpot analyses. The distribution of each dataset was evaluated via Anderson-Darling, D’Agostino–Pearson, and Shapiro-Wilk. Statistical significance was subsequently ascertained using a one-way ANOVA or two-way ANOVA, followed by a Tukey’s multiple comparison test when appropriate. Mann–Whitney–Wilcoxon-U test was used in all other instances. For both parametric and non-parametric tests, the significance threshold was set at *p* ≤ 0.05. For serum titer analyses, log_10_(EC_50_) were calculated from the dilution curve of each serum sample dilution, data are presented as means ± SEM. When only one serum dilution was analyzed, data are presented as means of optical densities at that dilution ± SEM.

### Reporting summary

Further information on research design is available in the Nature Portfolio Reporting Summary linked to this article.

## Results

Peptide immunogens targeting CGRP induce anti-CGRP antibodies in preclinical species

**Fig. 1:**
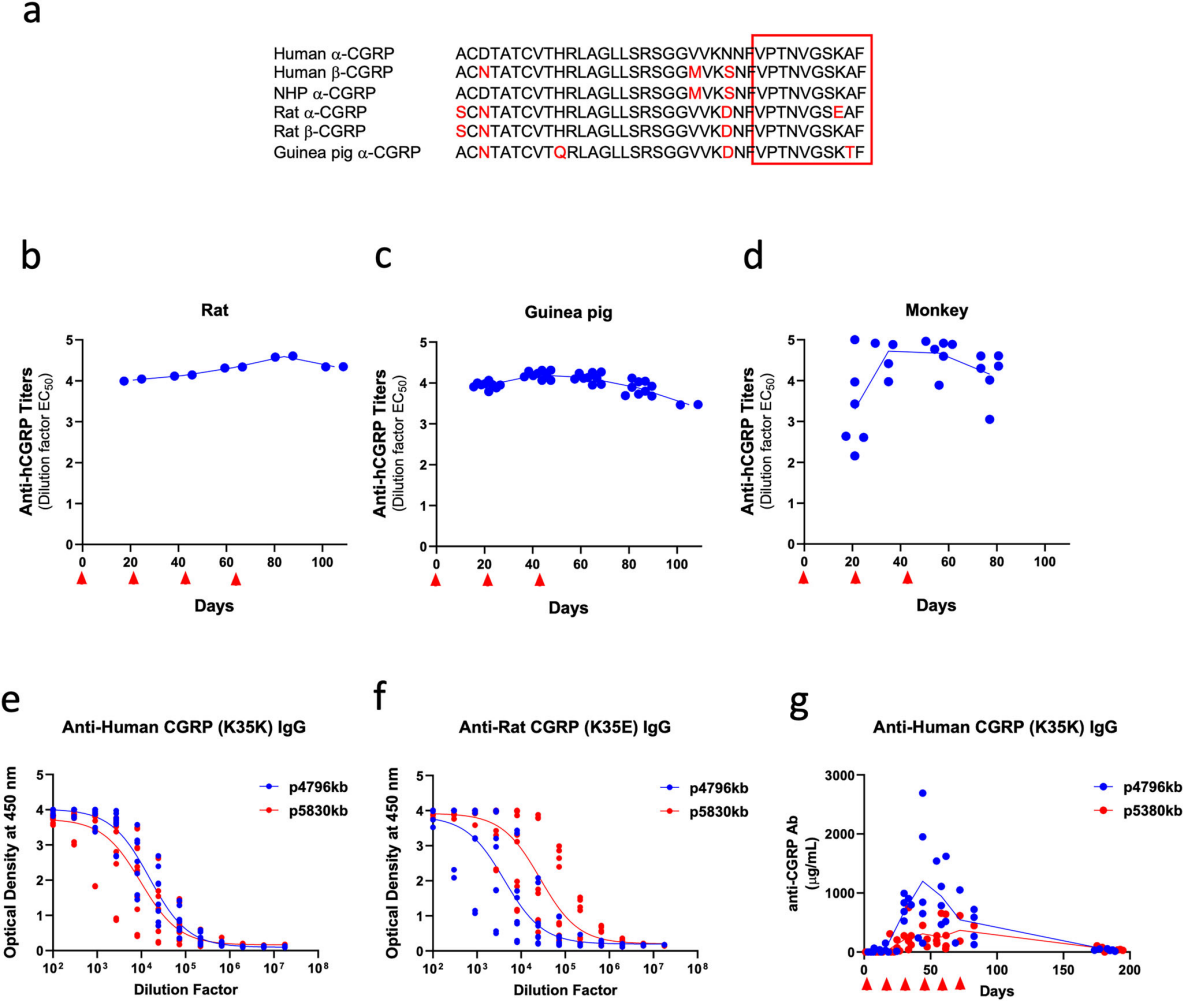
CGRP peptide immunogen induces high titers in rats, guinea pigs and monkeys. **a** Sequence alignment of the 37mer signaling peptide, CGRP. Conserved residues represented by *. Red box indicates the targeting peptide sequence (B cell epitope) of the human αCGRP peptide immunogen, p4796kb, and the rat αCGRP peptide immunogen, p5830kb. Anti-CGRP antibody titers over time in rat (*n* = 5) (**b**), guinea pig (*n* = 3) (**c**), and NHP (*n* = 3) (**d**). Anti-human αCGRP (K35K) (**e**) and anti-rat αCGRP (K35E) (**f**) IgG titers in sera collected post-immunization in rats injected with p4796kb or p5380kb (*n* = 5 per group). **g** Serum concentrations (μg/mL) of anti-CGRP antibodies. Red arrowheads indicate dosing time points. Individual data points are presented on each graph.

The mature 37-amino acid (aa) CGRP peptide is expressed as two major forms (αCGRP and βCGRP) and is well conserved across species, showing 90–95% homology to human αCGRP (Fig. 1a). The two forms of CGRP share similar biological functions, although their regional expression differs with αCGRP being primarily expressed in the central and peripheral nervous system whereas βCGRP is most abundantly expressed in the enteric nervous system. In humans, αCGRP and βCGRP differ by three amino acids, and in rats by only one. For NHP and guinea pigs, within each respective species, the sequences for αCGRP and βCGRP exhibit 100% homology. We designed a chimeric peptide immunogen where the C-terminus of CGRP (CGRP_28-37_) is linked to a promiscuous T_H_ peptide at the N-terminus via a poly-Lysine linker which constitutes the full peptide immunogen p4796kb. We first confirmed that this peptide was immunogenic across species (rats, guinea pigs, and nonhuman primates) by measuring anti-CGRP antibodies by ELISA, as illustrated in Fig. 1b–d. Importantly, although the C-terminal amide of α and β CGRP was not included in the peptide immunogen constructs, the commercial protein used to characterize the resultant peptide-derived antibodies contained this modification. From these results, we concluded that p4796kb can overcome immune tolerance across species, including in monkeys where there is a 100% sequence homology to humans. Importantly, the human CGRP sequence targeted by p4796kb contains a Lysine at residue 35 (K35K) while the rat sequence contains a glutamic acid at this residue (K35E). However, to ensure that we could also target rat αCGRP, we produced an additional peptide immunogen, p5830kb, that contains the same sequence as p4796kb with the exception of the single amino acid substitution (K35E). Rats immunized with either p4796kb or p5380kb produced comparable antibody titers against K35K CGRP as measured by enzyme-linked immunosorbent assay (ELISA) (Fig. 1e, Supplementary Fig. 1a, b). Interestingly, rats immunized with p4796kb showed lower titers against K35E CGRP than rats immunized with p5830kb (Fig. 1f), indicating that a single amino acid K35E substitution in the peptide immunogen can significantly impact the specificity of the antibodies. Nevertheless, evidence of anti-CGRP antibody titers among the species demonstrates that these peptide immunogens are immunogenic.

Next, we quantified serum concentrations of peptide-derived antibodies in rats immunized with p4796kb or p5830kb. Although the PK/PD relationship of peptide-derived antibodies is not directly comparable to a bolus of exogenous mAb, we investigated if immunization induces antibody serum concentration > 20 μg/mL, a level predicted to produce a pharmacodynamic response based on mAb treatment^12^. Moreover, we examined the durability of the antibodies by quantifying anti-CGRP antibodies more than three months after the last dose. Animals were dosed on Days 2, 16, 30, 44, 58, and 72, and sera were collected just before dosing and at day 184. No anti-CGRP antibodies were detected in rats treated with saline and adjuvant control. In rats immunized with either p4796kb or p5380kb, quantifiable levels of anti-CGRP antibodies were detected by Day 16 with maximal levels achieved between Days 30 and 44 (Fig. 1g). Serum antibody concentrations above 500 μg/mL and 200 μg/mL were achieved in rats immunized with p4796kb and p5380kb, respectively. While antibody concentrations seem to decrease during the course of immunization with p4796kb, no significant differences were noted between any of the time points at Days 30, 44, 58, and 72 (Tukey’s multiple comparisons test), which is consistent with the stable antibody titers observed during repeat immunization in other studies (Fig. 1b, Supplementary Fig. 1). Lower observed antibody concentrations in p5830kb-immunized rats might reflect faster target-mediated clearance with antibodies recognizing rat CGRP (K35E) and/or might be an artifact since the assay was optimized to measure antibodies against human CGRP (K35K). Although a direct comparison to the actual therapeutic concentrations achieved by mAbs could be misleading, we show here that immunization induces anti-CGRP antibodies at levels well above those predicted to be effective based upon mAbs. Finally, three months following the last dose, antibodies fell to 35.6 μg/mL and 48.8 μg/mL, indicating that discontinuation of dosing resulted in reduced serum antibody concentration and that repeat dosing with the peptide immunogen will be necessary for a sustained antibody response.

### IgG1 is the predominant isotype induced by immunization

**Fig. 2:**
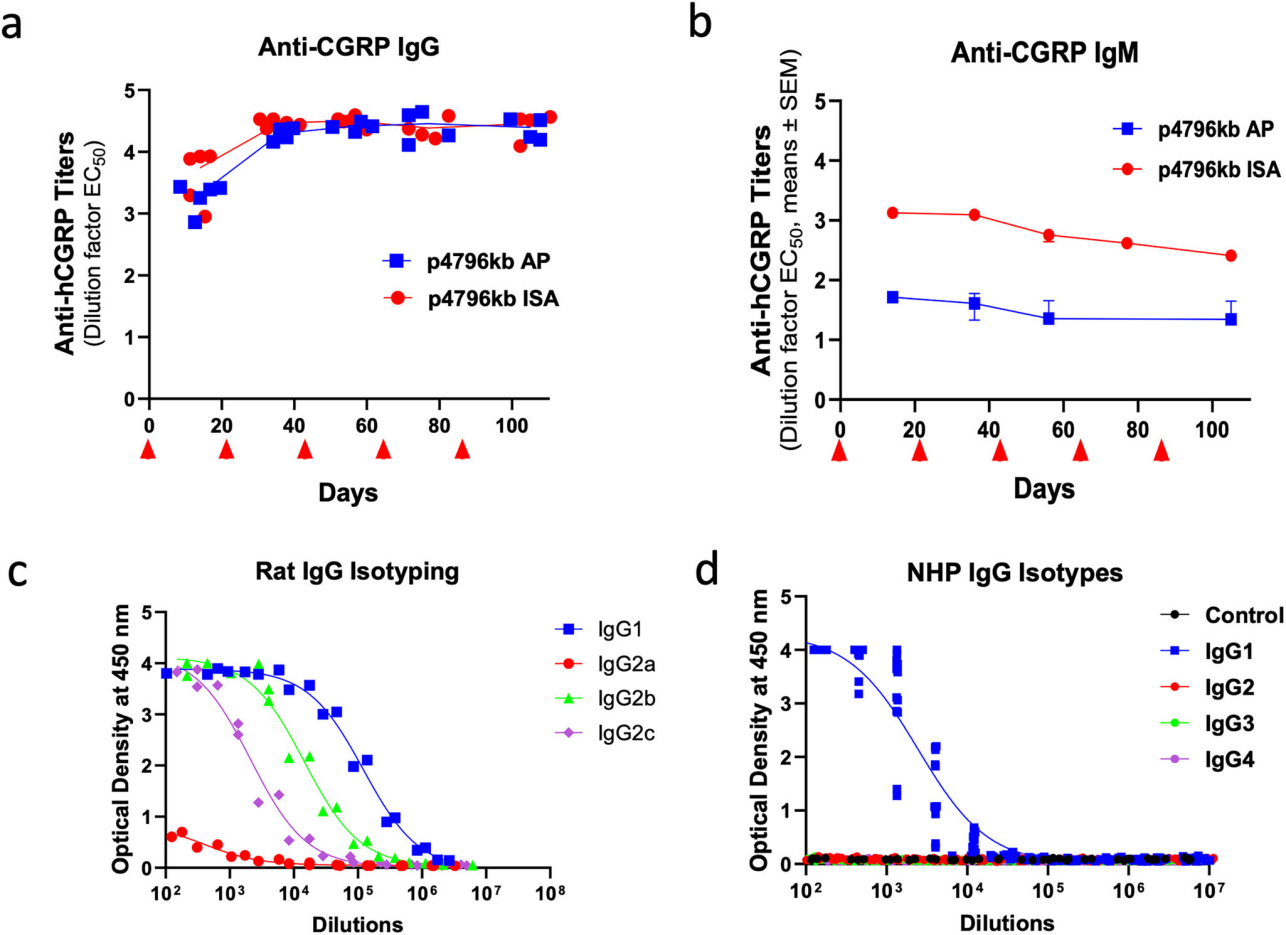
Immunization induces an IgG1 enriched response against CGRP. Anti-human αCGRP IgG (**a**) and IgM (**b**) titers in rats following immunization with p4796kb formulated in Adju-Phos^®^ (AP) or ISA-51VG (ISA) over time. **c** anti-CGRP IgG isotype titers from the terminal bleed of rats immunized with p4796kb formulated in Adju-Phos^®^. **d** Endpoint anti-CGRP IgG isotyping from monkeys injected with three doses three weeks apart of p4796kb formulated in Adju-Phos^®^. Red arrows indicate dosing time points. Individual data points are presented on each graph, except in (**b**) where data are presented as means +/− SEM. For rat study *n* = 5 per group. For NHP study *n* = 3 per group.

Because adjuvants can significantly alter the immune response to a peptide immunogen, we investigated how an Alum-based (Adju-Phos®) and Mineral Oil-based (Montanide™ISA-51VG) formulation would compare. In rats, peptides formulated in Adju-Phos® induced robust anti-CGRP IgG antibody titers with limited production of IgM against CGRP (Fig. 2a, b). In contrast, peptides formulated in ISA-51VG induced both IgG and IgM antibody titers (Fig. 2a, b). Further assessments of the IgG isotypes produced after immunization with an Alum-based formulation indicated a predominant production of IgG1 (Fig. 2c). Indeed, the IgG1 geometric mean EC_50_ titer (GMT-EC_50_) was 1:122,180 and was 308.3-fold higher than IgG2a (GMT-EC_50_ 1:308), 8.4-fold higher than IgG2b (GMT-EC_50_ 1:14,622) and 57.7-fold higher than IgG2c (GMT-EC_50_ 1:2118). However, using the same peptide immunogen formulated in ISA-51VG, the isotypes were more balanced than when using Adju-Phos® (Supplementary Fig. 1c). Notably, a study conducted in monkeys confirmed that immunization with p4796kb formulated in Adju-Phos® induced high titers of anti-CGRP IgG1, with no detectable CGRP-specific IgG2, IgG3 and IgG4 antibodies (Fig. 2d). Altogether these data confirm that the full peptide immunogens formulated in Adju-Phos® induces potent antibody responses enriched for IgG1 with minimum induction of IgM antibodies.

### Immunization has minimal effects on pro-inflammatory serum markers

We next assessed whether immunization against an endogenous peptide could be associated with inducing a general inflammatory response by assessing cytokine expression following dosing. Because the study was conducted in rats, we assessed the response following dosing with both p4796kb and p5380kb. A panel of 9 inflammatory cytokines (IFNγ, IL-1β, IL-4, IL-5, IL-6, IL-10, KC/Gro, and TNF-α) was measured in rat serum samples collected before immunization and 6 h after each dose. For each peptide immunogen, immunized rats showed cytokine levels within the range observed in adjuvant-treated rats (Supplementary Table 1). This evidence suggests that acute cytokine response following immunization is the result of adjuvant and not peptide components.

### The T helper peptide is required for T cell stimulation

**Fig. 3:**
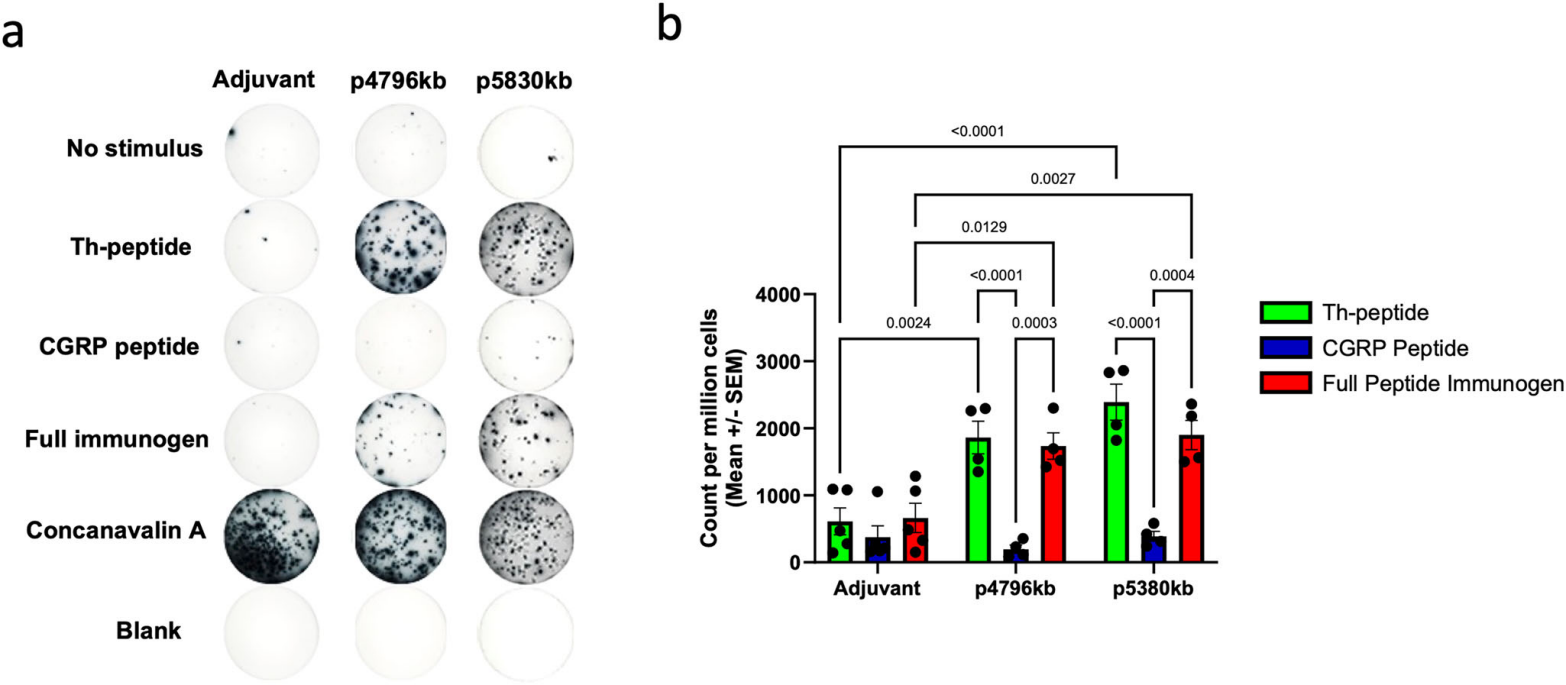
Splenocytes from immunized rats respond to the T_H_ peptide and full peptide immunogen, not to the CGRP peptide. **a** Representative image from ELISpot plate of IFNγ-secreting splenocytes collected from adjuvant control rats, p4796kb-immunized rats, or p5830kb-immunized rats. Splenocytes were either untreated or stimulated with the Th-peptide, CGRP, the full peptide immunogen, or the positive control Concanavalin A. Empty wells (Blank) are also included. **b** Quantification of spot count per million cells from the IFNγ ELISpot of splenocytes from rats immunized with p4796kb and p5380kb compared to Adjuvant control. Data are presented as means +/− SEM; *n* = 5 rats per group. *p < 0.05, **p < 0.01.

To confirm which peptide components induce the immune response, we evaluated splenocytes from immunized and naïve rats by measuring IFNγ expression using enzyme-linked immunosorbent spot (ELISpot) assays following exposure to the various peptide components. Splenocytes collected after immunization were stimulated with the CGRP peptide, the T_H_ peptide only, or with the full peptide immunogen. Control splenocytes from rats treated with only adjuvant did not respond to stimulation with any of the peptide components of the immunogen, strongly suggesting that splenocytes do not naturally react to these peptides. In contrast, both the T_H_ peptide and the full peptide immunogen induced activation as measured by the secretion of IFNγ from splenocytes collected after immunization (Fig. 3). Importantly, we observed that splenocytes do not react to the endogenous CGRP peptide, even after immunization, indicating that maintenance of antibody production will require re-exposure to the full immunogen construct. The ELISpot data demonstrates that both p4796kb and p5380kb require full peptide immunogen components to induce immune response without instructing the immune system to respond to endogenous CGRP.

### Immunization induces antibodies specific to the C-terminus of CGRP

**Fig. 4:**
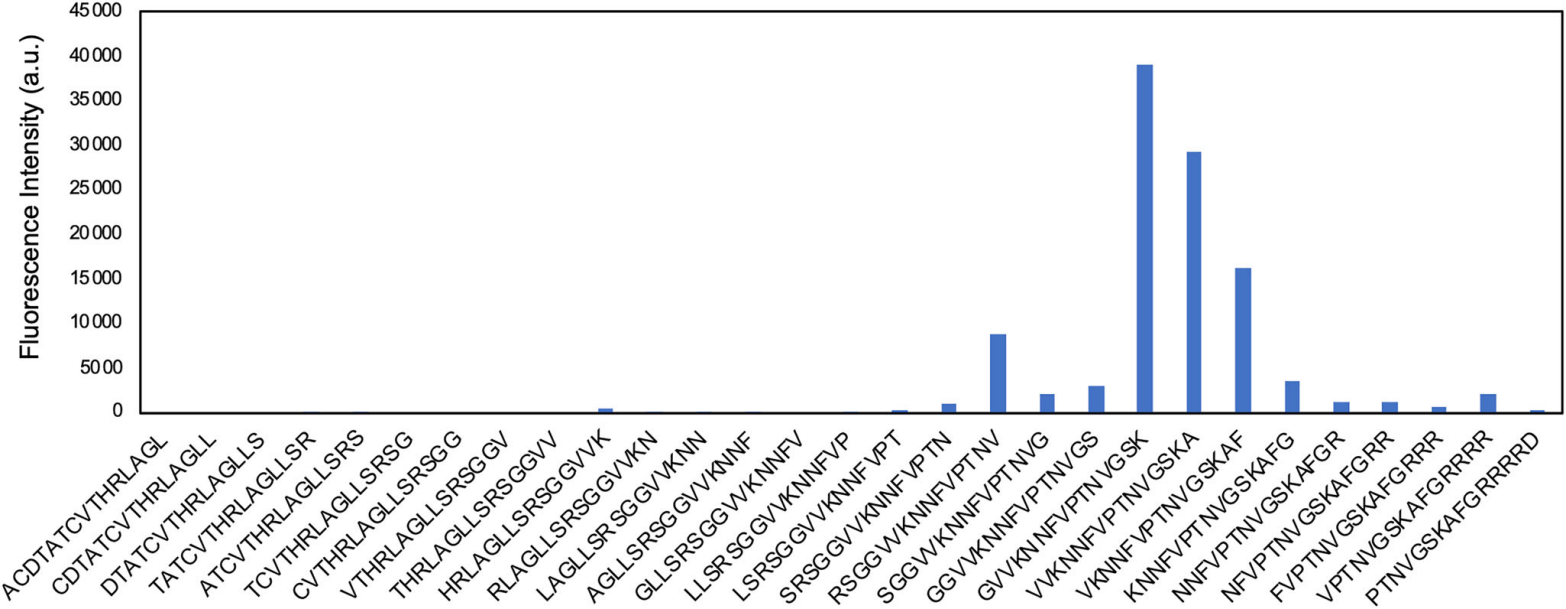
Immunization-derived antibodies bind to the C-terminal region of CGRP. Binding intensities of overlapping 15-mer peptides from N to C terminus of CGRP printed as an array. Plots are arranged from N to C terminal including poly arginine terminal extensions.

We designed the p4796kb CGRP peptide immunogen to target the C-terminal region of human CGRP, which is key for binding its receptor. To confirm the epitope targeted by the antibodies derived from this active immunotherapy, epitope mapping was conducted using the PEPperCHIP® platform. Briefly, anti-CGRP antibodies purified from sera of immunized Guinea pigs were applied to an array of overlapping amino acid sequences (15mers) representing the full sequence of human αCGRP. Incubation with anti-CGRP IgG to the peptide microarray confirmed that antibodies bind to the C-terminus of human αCGRP within the target peptide sequence (Fig. 4). Based on these results, we can conclude that the epitope targeted by the antibodies includes the three terminal amino acids, KAF. Importantly, the K35 residue seems to be critical for the recognition of human αCGRP (K35K) by p4796kb-derived antibodies, which could explain why rats immunized with this peptide immunogen demonstrate low binding or low titers against rat αCGRP (K35E).

Next, we examined the specificity of p4796kb-derived antibodies between αCGRP and βCGRP. As illustrated in Fig. 1a, there is perfect sequence alignment between human αCGRP and βCGRP to the targeted epitope. Thus, antibodies derived from p4796kb are not likely to distinguish between αCGRP and βCGRP. To confirm this, sera from guinea pigs immunized with p4796kb were used to examine the binding potency of antibodies to synthetic human αCGRP and βCGRP by dot blot analyses. At six- and nine-week post immunization, serum antibodies exhibit comparable signals against both αCGRP and βCGRP at equivalent dilutions with nine-week antibodies having a higher signal than six-week antibodies (Supplementary Fig. 2a). Consistent with the dot blot results, ELISA binding potencies of antibodies against αCGRP and βCGRP also indicated a lack of specificity between the two isoforms. Thus, both dot blot and ELISA analyses confirmed that the p4796kb-derived antibodies bind equivalently to synthetic human αCGRP and βCGRP (Supplementary Fig. 2b).

**Fig. 5:**
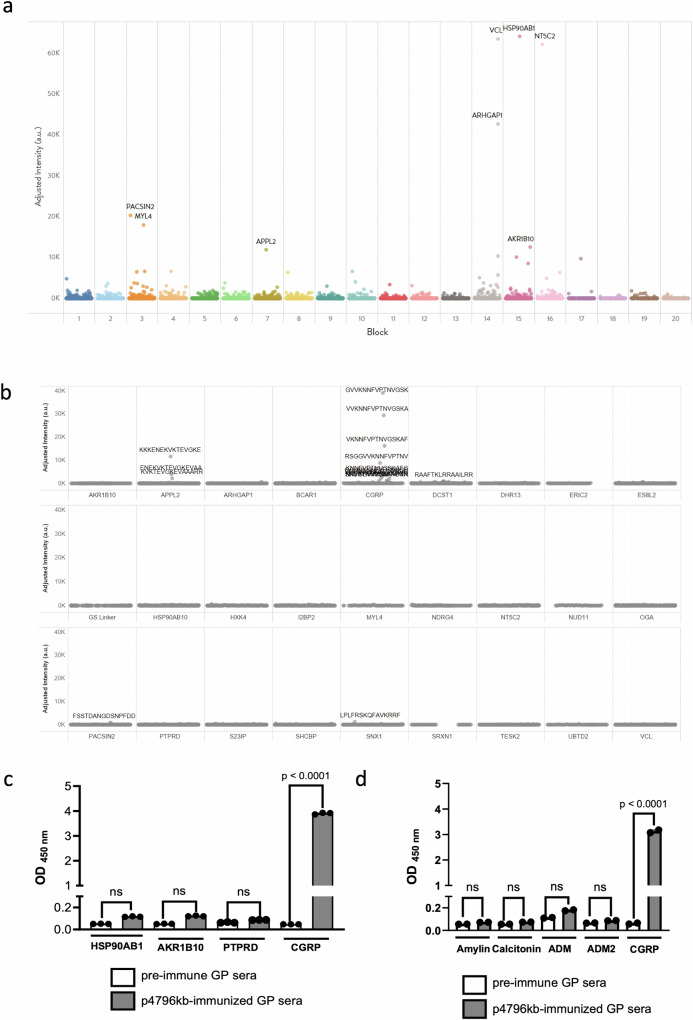
Immunization-induced antibodies are specific to CGRP. Proteome and peptide screens were used to evaluate potential off-target binding of affinity-purified p4796kb antibodies. **a** Manhattan plot displays results from the HuProt Human Proteome Microarray screen of antibody binding. Colors represent each block from the array, containing approximately 1000 proteins each. Spots represent individual protein intensity score from array. Hits are annotated with protein names. **b** Manhattan plot from peptide counter screen of 21 putative hits from proteome screen. Overlapping 15mer peptides were synthesized and printed on arrays. Spots represent individual peptide intensity scores from array. Peptide sequences of hits are displayed. Sera from guinea pigs immunized with p4796kb were tested for binding to putative hits from screens (**c**) and calcitonin family (**d**) measured by ELISA. Pre-immune sera were used as controls. Data are presented as means +/− SEM; *n* = 3. ***p < 0.0001.

We then examined the specificity of the antibodies generated after immunization with p4796kb to other human proteins and peptide targets. Sera from guinea pigs collected after immunization were assessed for potential off-target binding using the HuProt™ Human Proteome Microarray v4.0 against ~20,000 unique proteins (Fig. 5a). Top ranking hits are listed in Supplementary Table 2. The strongest putative interactions were assigned to proteins HSP90AB1 (heat shock protein 90 alpha family class B member 1), VCL (vinculin), and NT5C2 (5’-nucleotidase, cytosolic II). Weaker interactions were found with proteins ARHGAP1 (Rho GTPase activating protein 1), PACSIN2 (protein kinase C and casein kinase substrate in neurons 2), MYL4 (myosin light chain 4), AKR1B10 (aldo-keto reductase family 1 member B10), and APPL2 (adapter protein, phosphotyrosine). The potential hits were further evaluated by epitope mapping microarray (Fig. 5b). The intensity plot showed few responses with low to moderate intensities except for αCGRP. Only two of the hits from the proteome had signal above background. Sequences are included in Supplementary Table 3. ELISA-based binding assays were further conducted and demonstrated no binding to protein tyrosine phosphatase receptor type D (PTPRD) and only very weak binding to HSP90AB1 and AKR1B10 (Fig. 5c). Furthermore, we characterized the binding potential of p4796kb-derived antibodies to other propeptides that belong to the calcitonin/CGRP peptide family, including adrenomedullin (ADM), adrenomedullin 2 (ADM2), amylin, and calcitonin. No significant binding could be detected as compared to the binding to CGRP (Fig. 5d), further suggesting that p4796kb-derived antibodies are specific to CGRP.

### Immunization-derived antibodies are comparable to mAbs targeting CGRP

**Fig. 6:**
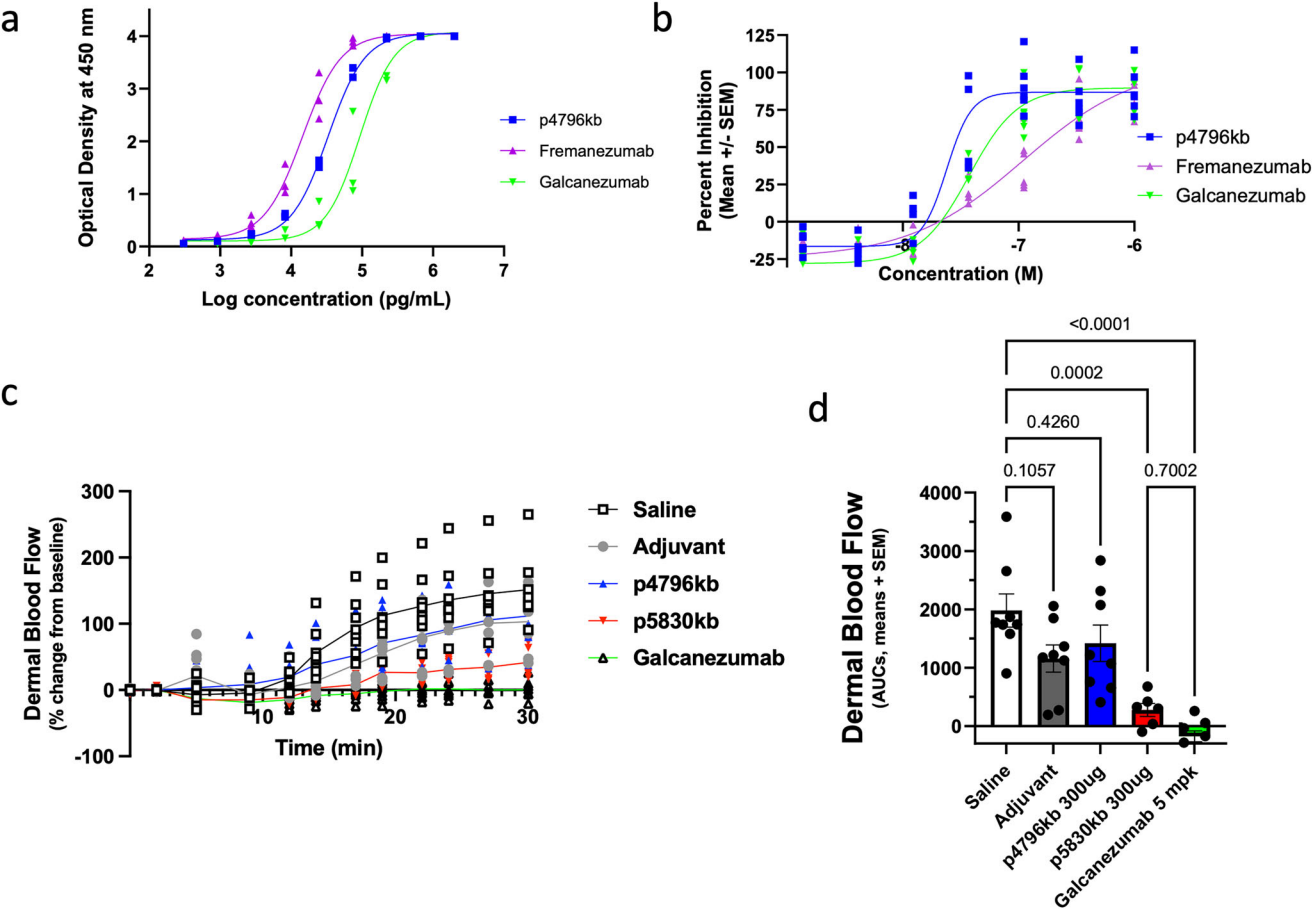
Immunization induces antibodies with properties comparable to mAbs. Affinity purified Abs from guinea pigs immunized with p4796kb were compared to the therapeutic mAbs, Galcanezumab and Fremanezumab, in binding potency (**a**) and in vitro activity assays (**b**). Inhibition of CGRP-induced cAMP stimulation in SK-N-MC. **c** Rats immunized with p4796kb and p5380kb were tested for capsaicin-induced dermal blood flow change over time. A two-way ANOVA indicated a significant treatment x time interaction (*p* < 0.0001), with p5830kb immunization significantly reducing the response to capsaicin (*p* < 0.0001), however, there was no significant effect of p4796kb immunization. **d** Area under the curve (AUC) analysis of percent dermal blood flow change; a one-way ANOVA confirmed a significant treatment effect (*p* < 0.0001); individual data points are presented on each graph and AUC data are also presented as means +/− SEM, *n* = 6 in the p5830kb group and *n* = 8 per group for all other treatment groups; *** *p* = 0.0002; **** *p* value ≤ 0.0001.

To confirm that the antibodies produced after immunization had the desired properties, we first characterized their binding potencies and affinities. Human αCGRP-specific IgG was affinity-purified from immunized guinea pig sera and compared to monoclonal antibodies, Fremanezumab and Galcanezumab for their binding kinetics by Surface Plasmon Resonance (SPR). Fremanezumab, Galcanezumab, and affinity purified antibodies had calculated equilibrium dissociation constants (*K*_*D*_) of 4.83 pM, 10.7 pM and 13.1 pM, respectively, with association rates of 3.59 × 10^5^ 1/Ms, 1.01 × 10^6^ 1/Ms vs. 3.50 × 10^6^ 1/Ms and dissociation rates of 1.73 × 10^−6^ 1/s, 1.08 × 10^−^^5^ 1/s vs 4.57 × 10^−^^5^ 1/s. Moreover, the affinity purified antibodies from p4796kb-immunized guinea pigs bound to human αCGRP with comparable potency to monoclonal antibodies in an ELISA binding assay (Fig. 6a). These experiments demonstrated that IgG produced after immunization with p4796kb bound to human αCGRP with affinity and potency in the range of a therapeutic mAb.

Additionally, we determined whether p4797kb-induced anti-CGRP antibodies could block the activity of CGRP to the same extent as Fremanezumab and Galcanezumab, in SK-N-MC cells using the Bridge-It® Cyclic AMP assay. Pre-treatment of the cell cultures with varying concentrations of anti-CGRP antibodies demonstrated a dose-dependent reduction of CGRP-induced cAMP (Fig. 6b). Affinity purified IgG derived from p4796kb immunization showed a similar in vitro activity (EC_50_ = 2.40 × 10^−8^M) to Galcanezumab (EC_50_ = 3.74 × 10^−8^M) and better activity than Fremanezumab (EC_50_ = 1.09 × 10^−7^M). The IgG fraction purified from pre-immune Guinea pig sera did not have detectable activities at all concentrations tested. These results indicate that CGRP-specific IgG induced by immunization inhibits CGRP signaling, comparable to a therapeutic anti-CGRP mAb.

### Immunization against K35E CGRP inhibits increased dermal blood flow induced by capsaicin in rats

Finally, we evaluated the functional effects of immunization in a pharmacodynamic rat model, the capsaicin-induced increase in dermal blood flow (DBF). Rats received repeat intramuscular injections of saline, Adju-Phos^®^, adjuvanted p4796kb, or adjuvanted p5830kb at 300 μg on Days 1, 22, 43, 64, and 85. An additional group of rats received a single intravenous infusion of Galcanezumab at 5 mg/kg on Day 85. Consequently, the CGRP epitope to which Galcanezumab binds (VTHRLAGLLSR) is the same between humans and rats. The capsaicin challenge was conducted 2 weeks after the 5th immunization or after Galcanezumab injection. The immunization regimen and timing of DBF testing were designed to ensure steady-state antibody titers and full affinity maturation was achieved before assessment. Antibody titers against human αCGRP (K35K) and rat αCGRP (K35E) were also evaluated during the study. In this study, rats receiving p4796kb again developed antibody titers ~100 times lower against K35E CGRP than against K35K CGRP. In contrast, rats receiving p5830kb developed similar antibody titers against K35K and K35E CGRP (Supplementary Fig. 3a, b). As expected, capsaicin induced a significant increase in dermal blood flow in rats immunized with saline or Adju-Phos^®^, an effect that was significantly altered by treatment (Treatment x Time interaction F_44_,_363_ = 11.14, *p* < 0.0001**;** Treatment effect F_4_,_33_ = 14.29, p < 0.0001, Fig. 6c). There was no significant effect of p4796kb immunization on dermal blood flow as compared to the saline and Adju-Phos^®^ groups. However, immunization with p5830kb significantly reduced the response to capsaicin when compared to the saline and Adju-Phos^®^ groups (Treatment x Time interaction: F_22_,_209_ = 8.989; *p* < 0.0001, Fig. 6c). Multi-comparison tests indicated a significant difference between p5830kb and Galcanezumab only at minutes 24 (p = 0.0326), 27 (*p* = 0.0206) and 30 (*p* = 0.0243) of the test session. Furthermore, analyses of the AUCs confirmed a significant effect of treatment (F_3_,_26_ = 25.11, *p* < 0.0001), with significantly lower AUCs in both p5830kb and Galcanezumab treatment groups compared to controls, and no significant differences between p5830kb and Galcanezumab (Fig. 6d). These results strongly suggest that the lack of effects of p4796kb, the peptide immunogen against human αCGRP (K35K), is due to the inferior binding of the antibodies to rat αCGRP (K35E). In contrast, rats immunized with p5830kb generated high antibody titers against rat αCGRP (K35E), which resulted in significant effects in the rat capsaicin challenge model.

## Discussion

Migraine is a prevalent and highly burdensome disease, with global prevalence of hundreds of million individuals^13,14^, and is the second most frequent cause of disability in the world^15^. The peak prevalence is observed in 25–55-year-old individuals, during the most productive time of life^16^. Multiple classes of medicines are available for the acute treatment of migraine, which provide variable relief across patients^17^. More recently, gepants, small molecules inhibiting CGRP receptor activation, and mAbs targeting CGRP have demonstrated significant efficacy in preventing episodic and chronic migraine. They are, however, only prescribed as second line therapies and are very expensive^18^. Thus, despite the various treatment options currently available, there remains a considerable unmet medical need for the prevention of migraine. Here we describe the properties of a peptide immunogen targeting CGRP that could potentially demonstrate similar efficacy as mAbs and provide patients with a more convenient and affordable prevention therapy option.

Our data indicate that it is possible to design a peptide immunogen that overcomes immune tolerance without inducing cytotoxic cell responses or chronic inflammation, and induces sufficient antibody titers to neutralize the intended target, CGRP. Attempts to design active immunotherapies against other endogenous proteins or peptides have so far achieved modest success, with several investigational active immunotherapies demonstrating acceptable safety and tolerability profiles, as well as immunogenicity, but have not yet demonstrated efficacy in the clinic^19,20^. However, this is to our knowledge the first active immunotherapy that was designed to target CGRP and that demonstrated a pharmacodynamic effect in a preclinical model.

To achieve a safe and efficient humoral response, the design of the peptide immunogen included a B cell epitope (CGRP C-terminus) linked to a promiscuous T_H_ peptide, which was formulated in an Alum-based adjuvant. The peptide immunogen formulated in Adju-Phos® demonstrated robust immunogenicity across several preclinical species and induced a predominant IgG1 response in rats and monkeys, while not inducing IgM antibodies. Additionally, no overt inflammation was observed by measuring circulating cytokines. Moreover, splenocytes collected after immunization only reacted to the peptide immunogen and the T_H_ peptide but did not respond to stimulation with high concentrations of the CGRP peptide. This strongly suggests that immune cells will not recognize the endogenous CGRP peptide as an antigen. This is also supported by the fact that serum antibody concentrations dramatically decrease over time in the absence of re-exposure to the peptide immunogen. Finally, no adverse events could be observed during in vivo studies across multiple species, and the GLP toxicity study indicated a favorable safety profile.

Characterization of the antibodies produced after immunization indicated strong binding potencies, binding kinetics, and activities in vitro, comparable to therapeutic mAbs. It is noteworthy that the in vitro properties observed with mAbs in our assays were comparable to the properties reported elsewhere^12^. Further characterization of the antibodies produced after immunization suggested that they were specific to CGRP but could not distinguish between α and β CGRP. Importantly, clinical data strongly suggest that long-term exposure to a mAb that does not distinguish between the two isoforms, such as Galcanezumab, is not associated with on-target safety or tolerability concerns. When investigating binding to other human proteins and peptides, a few potential off-target hits were identified in a comprehensive screen, but those hits could not be confirmed in our binding potency assays. As a limitation of the off-target screen that included a large repertoire of human recombinant proteins and/or synthetic peptides, we cannot exclude the possibility that some of the peptides or proteins included in the screen were not representative of the fully mature and physiological forms. However, our findings demonstrate that the peptide immunogen overcomes immune tolerance and induces antibodies most likely specific to endogenous CGRP.

We then demonstrated that the antibodies derived from anti-CGRP active immunotherapies are functional. In a cell-based assay, affinity purified anti-CGRP antibodies from immunized guinea pigs inhibited CGRP-mediated cAMP activation with comparable potencies to mAb. Additionally, immunization with p5830kb, the rat αCGRP targeted peptide immunogen, prevented the effects of local application of capsaicin on dermal blood flow in rats, a pharmacodynamic and translational model that has been extensively used to assess the effects of anti-CGRP drugs in preclinical models and in humans^11,21–23^. Interestingly, the peptide immunogen against human αCGRP was not effective in this pharmacodynamic model in rats, which could be attributed to a K35E substitution between the human and rat sequences at the C-terminus of CGRP. This observation highlights the high specificity of the peptide immunogen sequence and the need to design peptide immunogens with precision.

Since mAbs against CGRP have demonstrated breakthrough efficacy for the prevention of episodic and chronic migraine, a peptide immunogen designed to stimulate the production of endogenous antibodies against CGRP might provide an attractive option for the prevention of migraine. Monoclonal antibodies have a half-life less than a month, which requires that patients receive monthly injections of hundreds of milligrams of antibodies to manage migraine^14,24,25^. The high costs associated with the large-scale manufacturing of mAbs contribute to the very limited affordability and accessibility of these therapies. In contrast, peptide immunogens are highly scalable and are associated with low cost of goods, making them affordable for all patients around the globe. Furthermore, chronic treatment with mAbs may also activate an endogenous immune response that leads to anti-drug antibody (ADA)^24,26–28^. The ADA not only can bind to the therapeutic antibody and impact its efficacy but may also lead to the formation of immune complexes with undesirable side effects^29^. This issue is less of a concern for immunization-derived antibodies, because they are produced by one’s body, and the immune system is unlikely to produce antibodies against self-antibodies. Finally, as opposed to mAbs, an active immunotherapy is expected to provide the patient with a more convenient treatment regimen. Indeed, we have previously demonstrated that the antibody titers induced by UB-311 and UB-312, peptide immunogens with similar compositions to the CGRP peptide immunogen, have a half-life ranging from 8 to 12 weeks in humans^30^. This suggests that boosting the immune response may only be required quarterly or biannually.

In conclusion, we demonstrate that a peptide immunogen against CGRP induces potent antibodies against CGRP without inducing T-cell cytotoxicity or chronic inflammation. Based on the encouraging safety and activity profile of the active immunotherapy in preclinical species, as well as the data from a GLP toxicity study, UB-313 (p4796kb formulated in an Alum-based adjuvant) has advanced to clinical development (NCT05477095). If successful, this investigational active immunotherapy could provide a more convenient and affordable treatment option for patients with migraine. More generally, peptide immunogens may also represent an affordable and convenient strategy to prevent a broad range of diseases typically addressed by mAb therapies.

## Supplementary information

Supplementary Information

Description of Additional Supplementary Files

Supplementary Data 1

Reporting Summary

## Data Availability

The numerical data plotted (source data) in the graphs in Figs. 1, 2, 3, 5, 6, and Supplementary Figs. 1–3 are in Supplementary Data 1. Other data used in this publication will be made available to qualified researchers who provide a valid research question within the scope of the studies, and may be subject to a data use agreement. Requests will be responded to within 30 days. Please direct inquiries to the corresponding author (Jean-Cosme Dodart, jc@vaxxinity.com).

## References

[1] Disease, G. B. D., Injury, I. & Prevalence, C. Global, regional, and national incidence, prevalence, and years lived with disability for 328 diseases and injuries for 195 countries, 1990-2016: a systematic analysis for the Global Burden of Disease Study 2016. *Lancet* **390**, 1211–1259 (2017). 28919117 10.1016/S0140-6736(17)32154-2 PMC5605509

[2] Scuteri, D. et al. New trends in migraine pharmacology: targeting calcitonin gene-related peptide (CGRP) with monoclonal antibodies. *Front. Pharm.* **10**, 363 (2019). 10.3389/fphar.2019.00363 PMC6465320 31024319

[3] Bertels, Z. & Pradhan, A. A. A. Emerging treatment targets for migraine and other headaches. *Headache* **59**, 50–65 (2019). 31291018 10.1111/head.13585 PMC6986366

[4] Khan, K. et al. Analysis of treatment cost and persistence among migraineurs: a two-year retrospective cohort study in Pakistan. *PLoS One* **16**, e0248761 (2021). 33770109 10.1371/journal.pone.0248761 PMC7996986

[5] Kim, S. K., Nikolova, S. & Schwedt, T. J. Structural aberrations of the brain associated with migraine: a narrative review. *Headache* **61**, 1159–1179 (2021). 34407215 10.1111/head.14189

[6] Deen, M. et al. Blocking CGRP in migraine patients—a review of pros and cons. *J. Headache Pain.* **18**, 96 (2017). 28948500 10.1186/s10194-017-0807-1 PMC5612904

[7] Russell, F. A. et al. Calcitonin gene-related peptide: physiology and pathophysiology. *Physiol. Rev.* **94**, 1099–1142 (2014). 25287861 10.1152/physrev.00034.2013 PMC4187032

[8] Russo, A. F. & Hay, D. L. CGRP physiology, pharmacology, and therapeutic targets: migraine and beyond. *Physiol. Rev.* **103**, 1565–1644 (2023). 36454715 10.1152/physrev.00059.2021 PMC9988538

[9] Mavridis, T. et al. Monoclonal antibodies targeting cgrp: from clinical studies to real-world evidence-what do we know so far? *Pharmaceuticals*. **14**, 700 (2021). 10.3390/ph14070700 PMC8308667 34358126

[10] Yuan, H., Spare, N. M. & Silberstein, S. D. Targeting CGRP for the prevention of migraine and cluster headache: a narrative review. *Headache* **59**, 20–32 (2019). 31291020 10.1111/head.13583

[11] Benschop, R. J. et al. Development of a novel antibody to calcitonin gene-related peptide for the treatment of osteoarthritis-related pain. *Osteoarthr. Cartil.* **22**, 578–585 (2014). 10.1016/j.joca.2014.01.009 24508775

[12] Monteith, D. et al. Safety, tolerability, pharmacokinetics, and pharmacodynamics of the CGRP binding monoclonal antibody LY2951742 (galcanezumab) in healthy volunteers. *Front. Pharm.* **8**, 740 (2017). 10.3389/fphar.2017.00740 PMC5651004 29089894

[13] Monteith, T. S. Advocacy for migraine relief: strategic planning to eliminate the burden. *Curr. Pain. Headache Rep.* **26**, 567–574 (2022). 35716274 10.1007/s11916-022-01059-1 PMC9206221

[14] Edvinsson, L. CGRP and migraine: from bench to bedside. *Rev. Neurol.* **177**, 785–790 (2021). 34275653 10.1016/j.neurol.2021.06.003

[15] Steiner, T. J. & Stovner, L. J. Global epidemiology of migraine and its implications for public health and health policy. *Nat. Rev. Neurol.* **19**, 109–117 (2023). 36693999 10.1038/s41582-022-00763-1

[16] Lipton, R. B. & Bigal, M. E. The epidemiology of migraine. *Am. J. Med.* **118**, 3S–10S (2005). 15841882 10.1016/j.amjmed.2005.01.014

[17] Kung, D., Rodriguez, G. & Evans, R. Chronic migraine: diagnosis and management. *Neurol. Clin.* **41**, 141–159 (2023). 36400552 10.1016/j.ncl.2022.05.005

[18] Szok, D. et al. Chronic migraine as a primary chronic pain syndrome and recommended prophylactic therapeutic options: a literature review. *Life*. 13, 665 (2023). 10.3390/life13030665 PMC10056004 36983822

[19] Nimmo, J. T. et al. Amyloid-beta and alpha-synuclein immunotherapy: from experimental studies to clinical trials. *Front. Neurosci.* **15**, 733857 (2021). 34539340 10.3389/fnins.2021.733857 PMC8441015

[20] Zeitlinger, M. et al. A phase I study assessing the safety, tolerability, immunogenicity, and low-density lipoprotein cholesterol-lowering activity of immunotherapeutics targeting PCSK9. *Eur. J. Clin. Pharm.* **77**, 1473–1484 (2021). 10.1007/s00228-021-03149-2 PMC8440313 33969434

[21] Fiedler-Kelly, J. et al. Relationship of the calcitonin gene-related peptide monoclonal antibody galcanezumab pharmacokinetics and capsaicin-induced dermal blood flow in healthy subjects. *Clin. Pharm. Drug Dev.* **10**, 440–452 (2021). 10.1002/cpdd.929 33740315

[22] Hershey, J. C. et al. Investigation of the species selectivity of a nonpeptide CGRP receptor antagonist using a novel pharmacodynamic assay. *Regul. Pept.* **127**, 71–77 (2005). 15680472 10.1016/j.regpep.2004.10.010

[23] Buntinx, L., Vermeersch, S. & de Hoon, J. Development of anti-migraine therapeutics using the capsaicin-induced dermal blood flow model. *Br. J. Clin. Pharm.* **80**, 992–1000 (2015). 10.1111/bcp.12704 PMC4631172 26114340

[24] Kielbasa, W. & Helton, D. L. A new era for migraine: pharmacokinetic and pharmacodynamic insights into monoclonal antibodies with a focus on galcanezumab, an anti-CGRP antibody. *Cephalalgia* **39**, 1284–1297 (2019). 30917684 10.1177/0333102419840780 PMC6710614

[25] Moriarty, M. et al. Monoclonal antibodies to CGRP or its receptor for migraine prevention. *J. Nurse Pract.* **15**, 717–724.e1 (2019).

[26] Martinez, J. M. et al. Assessment of immunogenicity from galcanezumab phase 3 trials in patients with episodic or chronic migraine. *Cephalalgia* **40**, 978–989 (2020). 32340471 10.1177/0333102420920642 PMC7469706

[27] Chen, M. L., Nopsopon, T. & Akenroye, A. Incidence of anti-drug antibodies to monoclonal antibodies in asthma: a systematic review and meta-analysis. *J. Allergy Clin. Immunol. Pr.* **11**, 1475–1484.e20 (2023). 10.1016/j.jaip.2022.12.046 PMC10601343 36716995

[28] Mosch, R. & Guchelaar, H. J. Immunogenicity of monoclonal antibodies and the potential use of HLA haplotypes to predict vulnerable patients. *Front. Immunol.* **13**, 885672 (2022). 35784343 10.3389/fimmu.2022.885672 PMC9249215

[29] Johnson, D. E., Biotherapeutics: challenges and opportunities for predictive toxicology of monoclonal antibodies. *Int. J. Mol. Sci.* **19**, 3685 (2018). 10.3390/ijms19113685 PMC6274697 30469350

[30] Yu, H. J. et al. A randomized first-in-human study with UB-312, a UBITh(R) alpha-synuclein peptide vaccine. *Mov. Disord.* **37**, 1416–1424 (2022). 35426173 10.1002/mds.29016 PMC9545051

