# Supplementary material for: Preclinical characterization of an active immunotherapy targeting calcitonin gene-related peptide
Source: Commun Med (Lond). 2025 Apr 29;5:145. doi: 10.1038/s43856-025-00870-2 (PMC12041250; doi:10.1038/s43856-025-00870-2)

Supplementary information

Preclinical characterization of an active immunotherapy targeting Calcitonin gene-related peptide.

Justin D. Boyd^, Shixia Wang^, Hsiao-Wen Lin, Yueh-Ting Hsieh, Yu Shuang Sun, Brett A. Thibodeaux, Hanxin Lu, Jaya Sahni, Jonathan Wiggins, Matthew S. Longo, Jeanne K. Brooks, Madeline M. Vroom, Yi-Pin Chang, Zhi Liu, Shuang Ding, Jean-Cosme Dodart*

Supplementary Table 1: Summary of cytokines profiling data. Ranges from each group denote the low to high cytokine concentrations (in pg/mL) at any time point during the course of the study.

Cytokine (pg/mL)	Adjuvant	p4796kb	p5830kb
IFNγ	0.30 to 1.23	0.32 to 1.03	0.25 to 0.96
IL-10	2.32 to 4.90	1.98 to 3.92	2.31 to 4.25
IL-13	1.13 to 1.71	0.00 to 1.24	0.81 to 1.44
IL-1β	0.00 to 0.74	0.00 to 1.34	0.00 to 0.49
IL-4	0.64 to 0.93	0.55 to 0.96	0.55 to 0.85
IL-5	0.00 to 0.00	0.00 to 0.00	0.00 to 0.00
IL-6	2.34 to 18.13	0.60 to 12.80	1.92 to 32.91
KC/Gro	29.33 to 108.07	13.61 to 59.04	26.95 to 209.25
TNFa	0.74 to 2.06	0.72 to 1.19	0.83 to 1.75

Supplementary Table 2: Top 50 ranked proteins with spot intensities after staining with the p4796kb-immunized GP serum at 1:1000 dilution compared to pre-immune sera on HuProt™ Human Proteome Microarray.

Name	Preimmune, 1:1000	p4796kb, 1:1000	UniProt ID	RefSeq ID	Ensembl ID
HSP90AB1	1,335.0	65,378.0	P08238	NM_001271970	ENSP00000481908
VCL	0.0	63,480.0	P18206-2	NM_003373	ENSP00000211998
NT5C2	763.0	62,881.5	P49902	NM_001134373	ENSP00000339479
ARHGAP1	0.0	42,630.0	Q07960	NM_004308	ENSP00000432794
PACSIN2	687.5	20,927.0	Q9UNF0	NM_001184970	ENSP00000338379
MYL4	664.0	18,646.0	P12829	XM_005257391	ENSP00000347055
AKR1B10	647.0	13,086.0	O60218	NM_020299	ENSP00000352584
APPL2	0.0	11,825.0	Q8NEU8	NM_018171	ENSP00000447520
NUDT11	662.0	10,962.0	Q96G61	NM_018159	ENSP00000365160
PTPRD	694.0	10,687.5	P23468-5	XM_006716835	ENSP00000380741
SUPT5H	690.0	10,375.0	O00267	NM_001111020	ENSP00000384505
RSPH6A	681.5	9,143.0	Q9H0K4	NM_030785	ENSP00000472630
SDCCAG3	1,036.3	7,636.3	Q96C92-4	NM_006643	ENSP00000409357
MAGEA8	719.0	7,299.5	P43361	NM_001166400	ENSP00000443776
SPSB2	654.5	7,043.0	Q99619	NM_032641	ENSP00000428458
TFAP2D	0.0	6,496.0	Q7Z6R9	NM_172238	ENSP00000008391
SULT2B1	0.0	6,337.5	O00204	NM_177973	ENSP00000312880
TXLNB	722.5	6,330.0	Q8N3L3	XM_005266836	ENSP00000356624
PPP1R9A	0.0	6,288.5	Q9ULJ8	NM_017650	ENSP00000402893
ARMC9	712.5	5,762.0	Q7Z3E5-2	NM_025139	ENSP00000484241
PRR27	666.0	5,529.0	Q6MZM9	NM_214711	ENSP00000421773
C1QA	624.5	5,290.5	P02745	NM_015991	ENSP00000385564
TCAF1	881.0	4,896.0	Q9Y4C2	NM_014719	ENSP00000418432
HAGH	1,850.0	4,741.0	Q16775-2	NM_001040427	ENSP00000455355
HN1L	1,068.0	4,641.5	Q9H910	NM_144570	ENSP00000457344
SPRR3	1,612.0	4,565.5	Q9UBC9	NM_005416	ENSP00000330391
MAPK1IP1L	5,103.0	4,444.0	Q8NDC0	NM_144578	ENSP00000481882
PRRC2B	2,513.0	4,382.0	Q5JSZ5-2		
FAM175B	576.0	4,309.0		XM_011539554	
ANKRD40	588.0	4,166.0	Q6AI12	NM_052855	ENSP00000468442
FAM134B_frag	883.0	3,988.0			
SSB	629.5	3,976.0	P05455	NM_003142	ENSP00000386636
UBE2R2	785.5	3,848.0	Q712K3	NM_017811	ENSP00000263228
STX8	1,805.5	3,810.0	Q9UNK0	NM_004853	ENSP00000460073
QKI	0.0	3,749.2	Q96PU8	NM_006775	ENSP00000442848
TXNDC2	0.0	3,741.5	Q86VQ3-2	NM_001098529	ENSP00000483261
CLEC4M	650.0	3,673.5	Q9H2X3	NM_014257	ENSP00000351954
C1QTNF7	636.8	3,552.3	Q9BXJ2	NM_001135171	ENSP00000410722
SF3A2	689.5	3,399.0	Q15428	NM_007165	ENSP00000466634
EPM2AIP1	0.0	3,244.0	Q7L775	NM_014805	ENSP00000485091
TMSB10	630.0	3,192.5	P63313	NM_021103	ENSP00000233143
ALDH7A1	1,615.5	3,138.5	P49419-2	NM_001201377	ENSP00000414132
SCAMP5	658.0	3,072.0	Q8TAC9	XM_011521316	ENSP00000454602
NOL3	1,389.5	3,022.5	O60936-2	NM_001276312	ENSP00000268605
GAS8-AS1	800.0	2,941.0	O95177		
KCTD14	1,579.0	2,901.0	Q9BQ13-2	NM_001282406	ENSP00000481472
3-Sep	678.0	2,874.0			
PHPT1	0.0	2,844.5	Q9NRX4	NM_014172	ENSP00000247665
LBH	660.5	2,820.0	Q53QV2	NM_030915	ENSP00000386106
ATIC	1,094.0	2,780.0	P31939	NM_004044	ENSP00000401936

Supplementary Table 3: Summary of epitopes or peptide interactions against the preimmune serum or the p4796kb-immunized Guinea pig serum on an epitope mapping microarray.

Sample (Dilution)	Protein Hit from HuProt™ Protein Array	Epitope(s) from Epitope Mapping
Preimmune serum (1:1000)	-	-
p4796kb-immunized serum (1:1000)	CGRP (original antigen)	VPTNVGSK
	HSP90AB1	-
	VCL	-
	NT5C2	-
	ARHGAP1	-
	PACSIN2	TDANGDSNPFDD
	MYL4	-
	AKR1B10	-
	APPL2	KVKTEVGKE
	NUDT11	-
	PTPRD	-

Supplementary Figure 1: Immunization induces high anti-CGRP titers that are enriched in IgG1.

a,**b**, 1:300 sera dilution titers over time from rats immunized with p4796kb, p5830kb, and adjuvant only formulated in either Adju-Phos® (**a**) or ISA 51VG (**b**). **c**, Anti-CGRP IgG isotyping from rats immunized with p4796kb formulated in ISA. Red arrowheads indicate dosing time points. Data are represented as means +/- SEM, n = 5 per group.

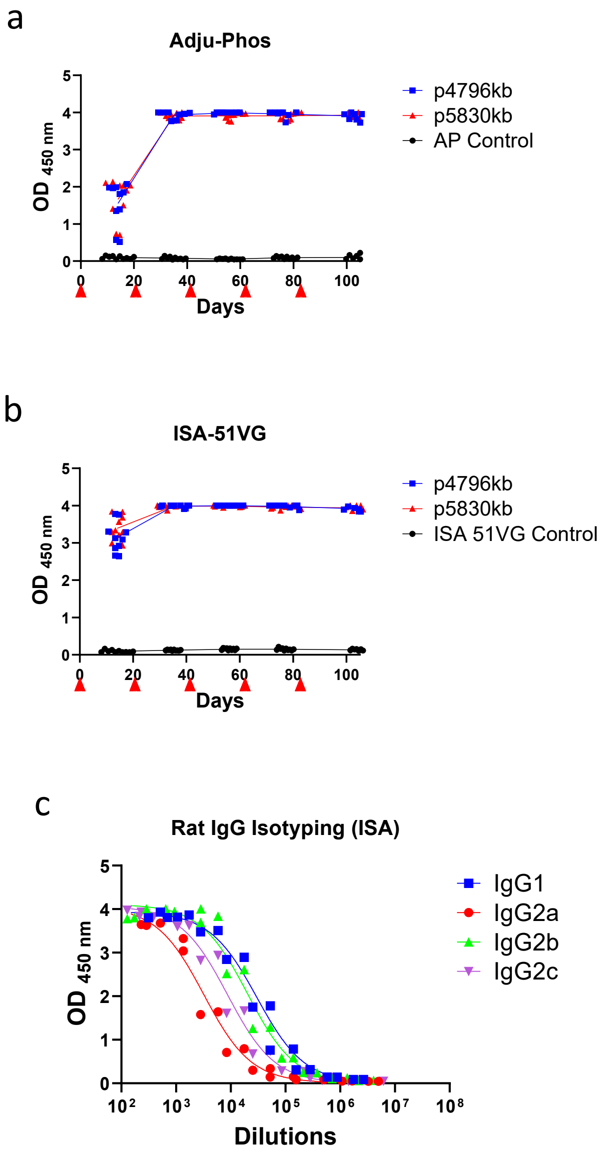

Supplementary Figure 2: p4796kb-derived antibodies bind to αCGRP and βCGRP.

a, b, Sera antibodies from guinea pigs immunized with p4796kb were used to compare the binding to full length synthetic human αCGRP and βCGRP. **a**, Dot blots showing binding of p4796kb derived antibodies to human αCGRP and βCGRP. Sera collected 6 and 9 weeks post injection (wpi) were applied in duplicates and at three dilutions to nitrocellulose membranes containing 200 ng of CGRP peptide per well. **b**. Comparable binding potency of antibodies to human αCGRP and βCGRP by ELISA. Data are presented as means +/- SEM, n = 3 per group.

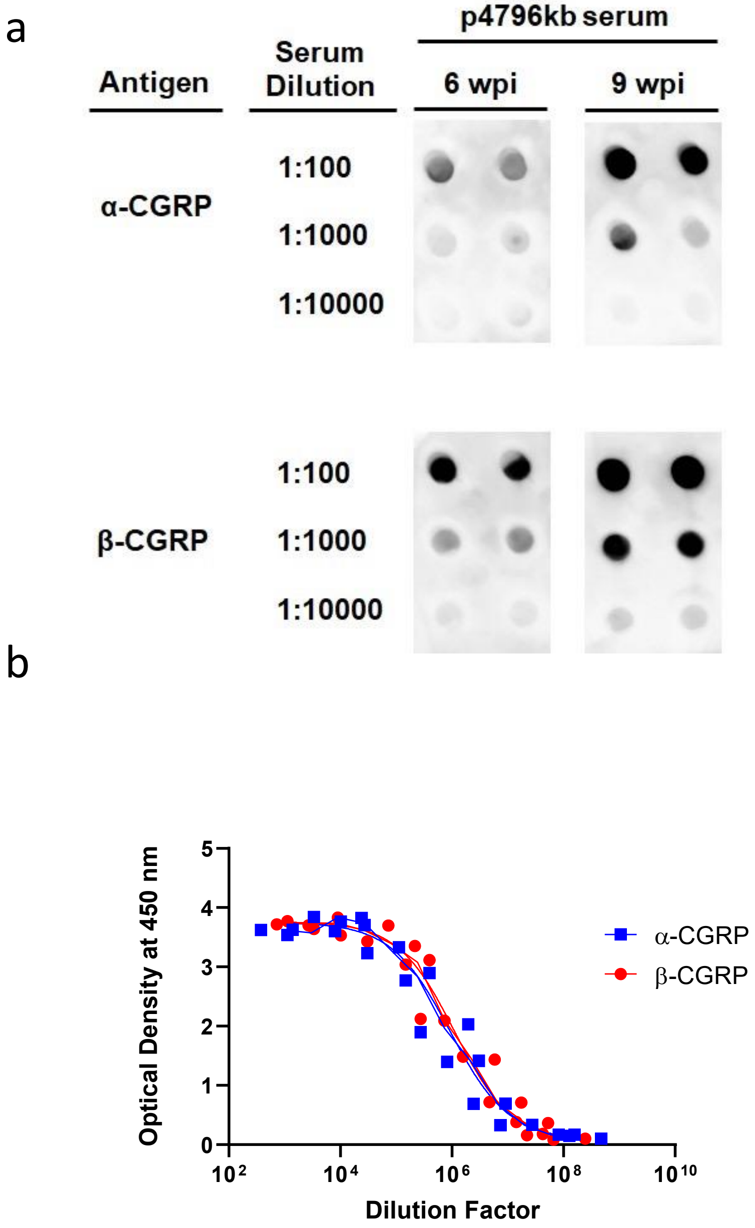

Supplementary Figure 3: p4796kb and p5380kb induce antibodies with differential binding potency for rat αCGRP (K35E) but not human αCGRP (K35K).

a,**b**, Rat IgG titers against K35K CGRP (**a**) and K35E CGRP (**b**) in rats immunized with p4796kb, p5380kb, Adju-Phos®, saline and Galcanezumab were analyzed in serum samples collected two weeks after the last injection (n = 8 per group). Data are presented as means +/- SEM, n = 3 per group.

a

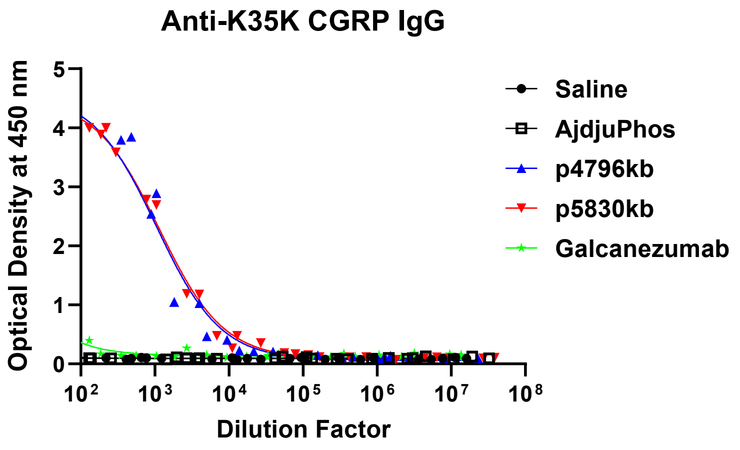

b

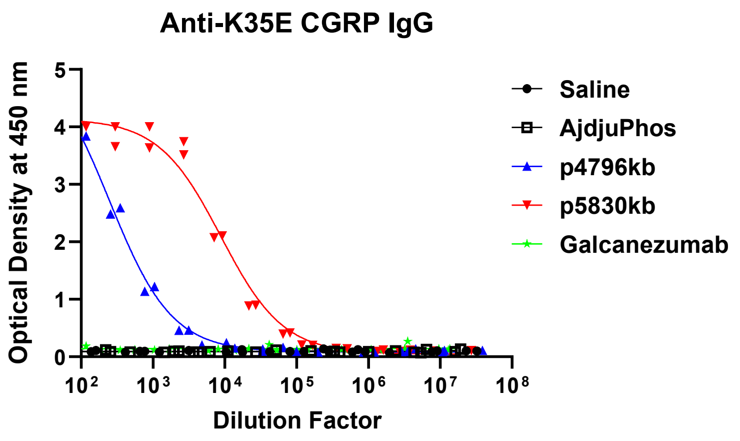

Supplementary Figure 4: Binding kinetic curves .

a, Binding kinetics of CGRP to antibodies. Antibodies were immobilized on a CM5 chip, a two-fold serial dilution of CGRP was sequentially injected at a flow rate of 30 μl/min for 120 seconds followed by dissociation for 600 seconds. The curves were fitted with Biacore®8K analysis software (BIAevaluation) using a 1:1 Langmuir binding model. **b**, Binding kinetics of CGRP to p4796kb-derived antibodies. Antibodies were immobilized on a CM5 chip, 200nM of CGRP was injected at a flow rate of 30 μl/min for 120 seconds followed by dissociation for 120 seconds.

a

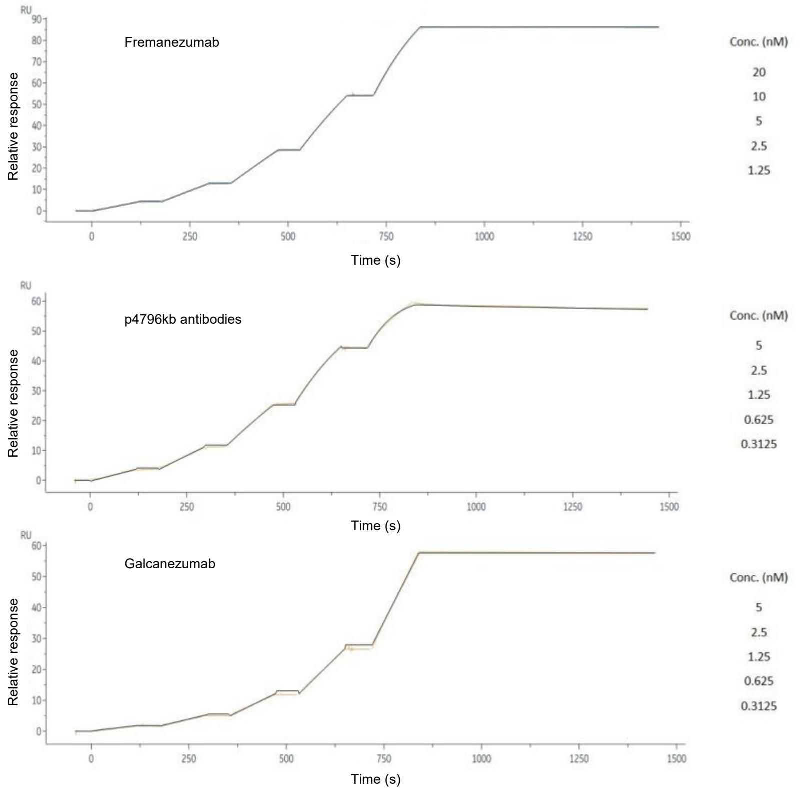

b

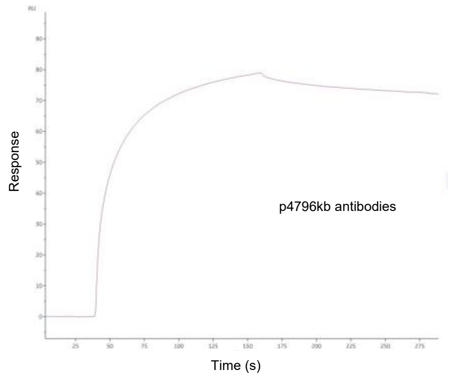

Supplement: Supplementary file 1 — Supplementary Information [file 43856_2025_870_MOESM1_ESM.pdf]
