# Supplementary material for: Preclinical characterization of an active immunotherapy targeting calcitonin gene-related peptide
Source: Commun Med (Lond). 2025 Apr 29;5:145. doi: 10.1038/s43856-025-00870-2 (PMC12041250; doi:10.1038/s43856-025-00870-2)
Supplement: Supplementary file 2 — Description of Additional Supplementary Files [file 43856_2025_870_MOESM2_ESM.pdf]

Description of Additional Supplementary Files

File name- Supplementary data 1

File description- The numerical data plotted (source data) in the graphs in Figures 1, 2, 3, 5, 6 and Supplementary Figures 1-3 is in Supplementary Data 1
