# Supplementary material for: Preclinical characterization of an active immunotherapy targeting calcitonin gene-related peptide
Source: Commun Med (Lond). 2025 Apr 29;5:145. doi: 10.1038/s43856-025-00870-2 (PMC12041250; doi:10.1038/s43856-025-00870-2)
Supplement: Supplementary file 4 — Reporting Summary [file 43856_2025_870_MOESM4_ESM.pdf]

nature portfolio

Corresponding author(s): Jean-Cosme Dodart

Last updated by author(s): Apr 6, 2025

Reporting Summary

Nature Portfolio wishes to improve the reproducibility of the work that we publish. This form provides structure for consistency and transparency in reporting. For further information on Nature Portfolio policies, see our Editorial Policies and the Editorial Policy Checklist.

Statistics

For all statistical analyses, confirm that the following items are present in the figure legend, table legend, main text, or Methods section.

n/a	Confirmed	
☐	☒	The exact sample size (*n*) for each experimental group/condition, given as a discrete number and unit of measurement
☐	☒	A statement on whether measurements were taken from distinct samples or whether the same sample was measured repeatedly
☐	☒	The statistical test(s) used AND whether they are one- or two-sided<br>*Only common tests should be described solely by name; describe more complex techniques in the Methods section.*
☒	☐	A description of all covariates tested
☒	☐	A description of any assumptions or corrections, such as tests of normality and adjustment for multiple comparisons
☐	☒	A full description of the statistical parameters including central tendency (e.g. means) or other basic estimates (e.g. regression coefficient) AND variation (e.g. standard deviation) or associated estimates of uncertainty (e.g. confidence intervals)
☐	☒	For null hypothesis testing, the test statistic (e.g. *F*, *t*, *r*) with confidence intervals, effect sizes, degrees of freedom and *P* value noted<br>*Give P values as exact values whenever suitable.*
☒	☐	For Bayesian analysis, information on the choice of priors and Markov chain Monte Carlo settings
☒	☐	For hierarchical and complex designs, identification of the appropriate level for tests and full reporting of outcomes
☒	☐	Estimates of effect sizes (e.g. Cohen's *d*, Pearson's *r*), indicating how they were calculated

Our web collection on statistics for biologists contains articles on many of the points above.

Software and code

Policy information about availability of computer code

Data collection	AID ELISpot Version 8.0 , SoftMax Pro 7.0
Data analysis	Microsoft Excel, GraphPad Prism 9, Tableau 2023.1, SoftMax Pro 7.0, EMBLEMBL-EBI MUSCLE alignment tool

For manuscripts utilizing custom algorithms or software that are central to the research but not yet described in published literature, software must be made available to editors and reviewers. We strongly encourage code deposition in a community repository (e.g. GitHub). See the Nature Portfolio guidelines for submitting code & software for further information.

Data

Policy information about availability of data

All manuscripts must include a data availability statement. This statement should provide the following information, where applicable:

- Accession codes, unique identifiers, or web links for publicly available datasets
- A description of any restrictions on data availability
- For clinical datasets or third party data, please ensure that the statement adheres to our policy

The source data for each graph can be found in the document "Source Data" in an Excel format.

Research involving human participants, their data, or biological material

Policy information about studies with human participants or human data. See also policy information about sex, gender (identity/presentation), and sexual orientation and race, ethnicity and racism.

Reporting on sex and gender	N/A
Reporting on race, ethnicity, or other socially relevant groupings	N/A
Population characteristics	N/A
Recruitment	N/A
Ethics oversight	N/A

Note that full information on the approval of the study protocol must also be provided in the manuscript.

Field-specific reporting

Please select the one below that is the best fit for your research. If you are not sure, read the appropriate sections before making your selection.

☒ Life sciences ☐ Behavioural & social sciences ☐ Ecological, evolutionary & environmental sciences

For a reference copy of the document with all sections, see nature.com/documents/nr-reporting-summary-flat.pdf

Life sciences study design

All studies must disclose on these points even when the disclosure is negative.

Sample size	In cases of guinea pigs (n = 3 per group) and NHP (n = 6 per group), cohort sizes were chosen to minimize the number of animals while providing statistical robustness. For rats, 6-8 animals per cohort were used to minimize the number of animals used while providing sufficient serum volume for antibody and cytokine characterization and splenocytes for an analysis.
Data exclusions	No data were excluded from analyses
Replication	For titers, replicates were collected within the same experiment and across experiments (at least one replicate experiment on a different day). In the case of isotyping and ELISpot experiments, two experiments were conducted by different individuals to confirm reproducibility. For in vitro and in vivo assays, "biological" replicates are presented. All other data are represented as triplicates. Data were confirmed to replicate.
Randomization	All studies involved randomization of animals or samples across treatment groups.
Blinding	All in vivo studies were conducted blinded. Some in vitro studies were not blinded, such as ELISpot experiments, to ensure adequate representation of each treatment group on each plate.

Reporting for specific materials, systems and methods

We require information from authors about some types of materials, experimental systems and methods used in many studies. Here, indicate whether each material, system or method listed is relevant to your study. If you are not sure if a list item applies to your research, read the appropriate section before selecting a response.

Materials & experimental systems

n/a	Involved in the study	
☐	☒	Antibodies
☐	☒	Eukaryotic cell lines
☒	☐	Palaeontology and archaeology
☐	☒	Animals and other organisms
☒	☐	Clinical data
☒	☐	Dual use research of concern
☒	☐	Plants

Methods

n/a	Involved in the study	
☒	☐	ChIP-seq
☒	☐	Flow cytometry
☒	☐	MRI-based neuroimaging

Antibodies

Antibodies used	Anti-CGRP mAbs Fremanezumab (Teva), Galcanezumab (Lilly), affinity purified anti-CGRP antibodies from rats, guinea pigs and NHPs immunized with our peptide vaccines (p4796kb and p5380kb).
Validation	mAb binding affinities were confirmed by biophysics (SPR) and were consistent with published data.

Eukaryotic cell lines

Policy information about cell lines and Sex and Gender in Research

Cell line source(s)	SK-N-MC cells(ATCC® HTB-10™), primary splenocytes were isolated directly from immunized rats
Authentication	cells were not authenticated by the investigators.
Mycoplasma contamination	cells were not tested for mycoplasma.
Commonly misidentified lines (See ICLAC register)	*Name any commonly misidentified cell lines used in the study and provide a rationale for their use.*

Animals and other research organisms

Policy information about studies involving animals; ARRIVE guidelines recommended for reporting animal research, and Sex and Gender in Research

Laboratory animals	Duncan-Hartley Guinea pigs (male,300-350g), Wistar rats(male,150-250g), Sprague Dawley rats(male,150-250g), Cynomolgus macaque (Macaca fasicularis, male, 25-50 months old, 2 to 5.5 kg)
Wild animals	*Provide details on animals observed in or captured in the field; report species and age where possible. Describe how animals were caught and transported and what happened to captive animals after the study (if killed, explain why and describe method; if released, say where and when) OR state that the study did not involve wild animals.*
Reporting on sex	Males were selected to eliminate the contribution of estrous cycle to treatment readouts.
Field-collected samples	*For laboratory work with field-collected samples, describe all relevant parameters such as housing, maintenance, temperature, photoperiod and end-of-experiment protocol OR state that the study did not involve samples collected from the field.*
Ethics oversight	All animal studies were conducted by contract research organizations (LabCorp or Charles River Laboratories), animals were bred and used in accordance with protocols approved by their internal Institutional Animal Care and Use Committee (IACUC).

Note that full information on the approval of the study protocol must also be provided in the manuscript.

Plants

Seed stocks	*Report on the source of all seed stocks or other plant material used. If applicable, state the seed stock centre and catalogue number. If plant specimens were collected from the field, describe the collection location, date and sampling procedures.*
Novel plant genotypes	*Describe the methods by which all novel plant genotypes were produced. This includes those generated by transgenic approaches, gene editing, chemical/radiation-based mutagenesis and hybridization. For transgenic lines, describe the transformation method, the number of independent lines analyzed and the generation upon which experiments were performed. For gene-edited lines, describe the editor used, the endogenous sequence targeted for editing, the targeting guide RNA sequence (if applicable) and how the editor was applied.*
Authentication	*Describe any authentication procedures for each seed stock used or novel genotype generated. Describe any experiments used to assess the effect of a mutation and, where applicable, how potential secondary effects (e.g. second site T-DNA insertions, mosiacism, off-target gene editing) were examined.*
